# Relative Clinical Efficacy and Safety of Second- or Later-Line Treatments for Advanced and Metastatic Gastric Cancer: A Rapid Review and Network Meta-Analysis

**DOI:** 10.1007/s12029-026-01407-z

**Published:** 2026-02-06

**Authors:** Shikha Sharma, David McConnell, Niamh Carey, Jacintha O’Sullivan, Patrick Kearns, Maeve Lowery, Laura McCullagh

**Affiliations:** 1 Trinity College Dublin, Dublin, Ireland; 2 National Centre for Pharmacoeconomics, Dublin, Ireland; 3 St James’s Hospital, Dublin, Ireland

**Keywords:** Gastric cancer, Advanced and metastatic, Systemic treatments, Evidence synthesis

## Abstract

**Objective:**

To identify randomised control trials (RCTs) of treatments (recommended by the National Comprehensive Cancer Network (NCCN), the European Society for Medical Oncology (ESMO) Clinical Practice Guidelines and clinical expertise) for the second- or later-line treatment of advanced/metastatic gastric cancer. To determine the relative efficacy and safety of the treatments.

**Methods:**

RCTs were identified from a Rapid Literature Review and a published systematic review. Identified RCTs were subject to data-extraction and narrative review. Eligible RCTs were included in evidence networks to determine relative efficacy and safety of the treatments.

**Results:**

In total, 44 RCTs (pertaining to eleven treatments), were identified for data-extraction and narrative review; 37 in the second-line setting, five in the second- and later-line setting and two in the third- and later-line setting. Evidence networks were feasible for the second-line treatments only. No statistically significant differences, across treatments, for key efficacy outcomes (overall-survival, progression-free survival), and additional outcome (objective-response rate) were identified. Pembrolizumab was associated with a statistically significant decreased risk of Grade ≥ 3 treatment-related adverse effects versus paclitaxel; no other significant differences, across treatments, were identified for this outcome.

**Conclusion:**

The appreciable number of RCTs identified indicates that the treatment landscape here is rapidly evolving. The introduction of novel treatments, in the second-line setting, has not had a statistically significant impact on key efficacy outcomes, and has had little impact on safety outcomes, versus more established treatments. There remains a need for novel treatments that will have a significant benefit on efficacy and safety outcomes.

**Supplementary Information:**

The online version contains supplementary material available at 10.1007/s12029-026-01407-z.

## Introduction

According to GLOBOCAN estimates, in 2022, gastric cancer ranked fifth, globally, in incidence rate and was the fifth most frequent cause of cancer-related mortality. At that time, over one million individuals, across 185 countries, had advanced or metastatic gastric cancer [1]. The estimated 5-year relative survival rates for localised, regional, and distant or metastatic gastric cancers in the US, for 2014 to 2020, were 75%, 36% and 7%, respectively [2]. While curative surgical or endoscopic resection are the cornerstone treatments for early stages of gastric cancer, systemic treatments are the standard of care in the advanced setting [3].

Recent advances in molecular profiling of gastric adenocarcinomas have identified key subtypes. These include Epstein Barr virus (EBV) positive, microsatellite instability-high (MSI-H), genomically stable, and tumours with chromosomal instability (CIN) [4]. CIN subtypes show copy number variations in key oncogenes such as epidermal growth factor receptor (EGFR), human epidermal growth factor receptor 2 (HER-2), fibroblast growth factor receptor 2 (FGFR2) and mesenchymal-epithelial transition factor (MET) [4, 5].

There have been recent advances in the use of biomarker-directed therapies [6]. For example, the European Society for Medical Oncology (ESMO) Clinical Practice Guidelines 2022 recommend programmed cell death protein 1 (PD-1) inhibitors for programmed death-ligand 1 (PD-L1) positive tumours where combined positive scores (CPS) determine the eligibility for immune checkpoint inhibitors (ICIs) [6, 7]. In metastatic gastric cancer, Microsatellite Instability-high/Deficient (MSI-H)/Mismatch Repair deficiency (dMMR) is associated with increased sensitivity to immunotherapy [8]. Ongoing trials are examining the efficacy and safety of other biomarker-targeted therapies, such as those for use in the settings of FGFR2b overexpression [9, 10], MET amplification, claudin-18.2 overexpression and EBV-positive cancers [7], and novel HER-2 directed therapies alone and in combination with immune checkpoint blockade [11].

Most individuals diagnosed with gastric cancer present with advanced-stage disease, often resulting in poor outcomes [12]. Common first-line chemotherapies include fluoropyrimidine in combination with platinum compounds (fluoropyrimidine + platinum compounds) [7]. Oxaliplatin and cisplatin are amongst the commonly utilized platinum compounds [7]; oxaliplatin is preferred for older adults [13]. Fluoropyrimidines are given either intravenously (5-FU) or orally (capecitabine or S-1 (which is more commonly used in Asia)) [7, 14]. 5-FU + platinum compounds + taxane is not recommended as a standard approach due to high toxicity [7] but may be considered in the setting of oligometastatic disease [15]. In cases of platinum-intolerance, individuals may be given 5-FU monotherapy or 5-FU + irinotecan. HER-2 overexpression may be treated with trastuzumab (a monoclonal antibody, specifically targeted against the HER-2 extracellular domain) + chemotherapy [7] with pembrolizumab added in the setting of PD-L1 (CPS) ≥ 1 [16, 17]. Where the HER-2 status is negative with PD-L1 (CPS) ≥ 5, nivolumab + chemotherapy is recommended [7]. Pembrolizumab + chemotherapy is approved regardless of PD-L1 (CPS) status [18]. Zolbetuximab (a claudin-18.2 directed cytolytic antibody), is approved, in combination with fluoropyrimidine + platinum compounds, for first-line treatment of individuals with claudin-18.2-positive and HER-2 negative tumours [19, 20]. Treatment options, in the first-line treatment setting, are limited. Survival outcomes are poor following disease progression on first-line treatments [21].

Thus, over recent years, there have been rapid changes in the treatment landscapes in the second- and later-line settings [6]. Notably, in the developments of immunotherapies and targeted therapies.

The ESMO and NCCN guidelines generally recommend similar second-line chemotherapy options, but the guidelines differ in some recommendations regarding novel treatments and the sequencing of treatments. In summary, the ESMO guidelines recommend that individuals without contraindications to chemotherapy or anti-angiogenic treatment should receive ramucirumab + paclitaxel. ESMO recommends that individuals who are unable to tolerate chemotherapy should receive ramucirumab monotherapy and those who are unable to tolerate anti-angiogenic treatments should receive a taxane or irinotecan monotherapy [7]. FOLFIRI (5-FU + leucovorin + irinotecan) is recognised by ESMO as an alternative option, although the supporting evidence is limited [7]. FOLFIRI, however, is listed as a preferred option in the NCCN guidelines [22]. Other NCCN recommended preferred options are ramucirumab + paclitaxel, docetaxel monotherapy, paclitaxel monotherapy or irinotecan monotherapy [22]. With regards to immunotherapy, both ESMO and NCCN recommend pembrolizumab in the setting of MSI-high (MSI-H) or mismatch repair deficient (dMMR) tumors [7, 22]. In addition, NCCN also recommends pembrolizumab if tumor mutational burden is ≥ 10/megabase, dostarlimab as another option for MSI-H/dMMR tumors and entrectinib in individuals with NTRK gene fusion positive tumors [22]. Both guidelines recommend trastuzumab deruxtecan for HER2-positive tumors in the second- and later-line settings [7, 22]. The ESMO guidelines recommend trifluridine–tipiracil as a third-line treatment [7]; the NCCN recommends trifluridine–tipiracil in the third or later-line setting ’ [22].

Of interest here, Catenacci et al. [23], conducted a systematic literature review (SLR) of randomised clinical trials (RCTs) (published 2009 to 19 November 2019 inclusive), that had investigated the efficacy and safety of systemic treatment, for adults with advanced, unresectable or metastatic gastric cancer, or gastroesophageal junction adenocarcinoma. Amongst their findings, the authors concluded that sequencing with taxanes or irinotecan, in combination with biologics, were effective second-line regimens. It was also noted that escalating to a triplet regimen might improve treatment efficacy at the expense of added toxicity [23].

It is recognised that, despite the survival benefits associated with novel second-line treatments, the prognosis here continues to be poor [24]. Also, there remains much uncertainty regarding the relative efficacy and safety of these novel treatments. This presents challenges for health-decision makers, including policy-decision makers, health-payers, clinicians, patients, carers and families.

The objective of this research is to examine the changing landscape of treatments of interest for advanced and metastatic gastric cancer in the second- or later-line setting. Also, to investigate the relative efficacy and safety of these treatments. Treatments of interest were defined as those recommended by both the ESMO Guidelines [7] and the NCCN Guidelines [22] and also that align with Clinical Expert opinion.

To capture the relevant literature, the search conducted as part of this research aimed to identify the published RCTs of treatments of interest. Those studies that had been identified by Catenacci et al. were reviewed for eligibility. In addition, a Rapid Literature Review (RLR) was undertaken to identify RCTs that had been published after the Catenacci et al. dissemination. An RLR is a recognised, pragmatic approach used to identify literature of relevance to a research question. In order to estimate the relative efficacy and safety of the treatments of interest, network evidence syntheses were conducted, in the form of network meta-analyses (NMAs), of eligible RCTs.

## Methods

### Identification of Treatments of Interest

The PICOS Framework for the literature search is presented in Appendix (Table 1). The population of interest was individuals with advanced or metastatic gastric cancer in the second- or later-line setting.

The ESMO Guidelines (Lordick et al., 2022) and NCCN Guidelines (Ajani et al., 2022) were systematically reviewed to identify treatments of interest [7, 22]. In compliance with both of these guidelines, RCTs that evaluated at least one of the following treatments: paclitaxel, docetaxel, irinotecan, FOLFIRI, ramucirumab, ramucirumab + paclitaxel, pembrolizumab (in the setting of MSI-H/dMMR tumours), dostarlimab, entrectinib, trastuzumab deruxtecan (in the setting of HER-2 positive tumours) and best supportive care (BSC) were considered for inclusion. Further, the NCCN Guidelines, also recommends pembrolizumab in the setting of TMB-high tumours.

Clinical Expertise advised that all treatments, on this list, should be included along with trifluridine-tipiracil for third- and fourth-line treatment.

### Search Strategy for Eligible Randomised Controlled Trials

The two-step search strategy aimed to identify eligible RCTs published from 2009 to 29 May 2024 inclusive.

#### Step One

A comprehensive RLR for eligible RCTs (published 20 November 2019 to 29 May 2024 inclusive), was conducted. A systematic search of Cochrane Central, Embase, and MEDLINE via Ovid was performed. The search string is presented in Appendix 1. The RLR was restricted to English-language, full-text publications. Conference papers, commentaries, abstracts, and letters were excluded. Subgroup, exploratory and post-hoc analyses of original trials were also excluded. Further information regarding our inclusion criteria is provided in Table 1.

**Table 1:** Randomised controlled trials included in data-extraction & narrative review

Trial	Key Trial & Population characteristics	Interventions	Overall Survival		Progression Free Survival		Objective Response Rate		Grade ≥ 3 Treatment-Related Adverse Events	
			Median (95% CI), months	Hazard Ratio (95% CI)	Median (95% CI), months	Hazard Ratio (95% CI)	Absolute Number (*n*) Rate (%) 95% CI	Relative Risk (95% CI)	Absolute number (Rate: 95% CI) (Safety data)	Relative Risk (95% CI)
*Second-line Setting (n = 37)*										
Paclitaxel versus Paclitaxel in Combination with Various Treatments										
Shah et al. (2022) [70]	Phase III Median follow-up: 6.8 months Location: Asia, North America, Europe, Australia, South America Prior Treatment: Fluoropyrimidine + platinum compounds	Napabucasin + Paclitaxel *n* = 357 Median age years (range) 63.1 (25.4–86.2) Males *n* = 261 (73.1%)	6.93 (6.28–7.69)	1.01 (0.86–1.20) *P* = 0.86	3.55 (3.22–3.68)	1.01 (0.85–1.19) *P* = 0.90	n = NR Rate = 16% (95%CI = NR)	NR	*n* = 112 Rate = 31.40% (95%CI = NR)	NR
		Paclitaxel *n* = 357 Median age years (range) 61.7 (24.1–88.0) Males n = 254 (71.1%)	7.36 (6.64–8.15)		3.68 (3.48–3.71)		n = NR Rate = 18% (95%CI = NR)		*n* = 37 Rate = 10.60% (95%CI = NR)	
Zhao et al. (2023) [80]	Phase II Median follow-up: 13 months Median age years (range) 55.3 (25–74). Males *n* = 87 (63.5%) Location: China Prior Treatment: Fluoropyrimidine + platinum compounds + trastuzumab (in the HER-2 positive setting)	Raltitrexed + Paclitaxel *n* = 73	NR	NR	NR	NR	*n* = 5 Rate = 6.8% (95%CI = NR)	NR	n = NR Rate = 60.60% (95%CI = NR)	NR
		Paclitaxel *n* = 75					*n* = 3 Rate = 4.0% (95%CI = NR) *P* = 0.72		n = NR Rate = 57.50% (95%CI = NR)	
Makiyama et al. (2020) [48]	Phase II Median follow-up: 10 months Location: 52 centres in Japan Prior Treatment: Fluoropyrimidine + platinum compounds	Paclitaxel *n* = 46 Median age years (range): 67 (33–81) Males *n* = 39 (86.7%)	10.0 (7.6–13.1)	1.23 (0.76–1.99) *P* = 0.20	3.2 (2.9–3.5)	0.91; 80% CI, (0.67–1.22) *P* = 0.3	n = NR Rate = 32.0% (95%CI = 17.5–48.7)	NR	NR	NR
		Paclitaxel + Trastuzumab *n* = 45 Median age years (range): 65 (50–89) Males *n* = 32 (72.7%)	10.2 (7.9–12.8)		3.7 (2.8–4.5)		n = NR Rate = 33.0% (95%CI = 19.1–50.2) *P* = 1.0		NR	
Bang et al. (2015) [58]	Phase II Median follow-up: 8.4 months Location: Korea Prior Treatment: Fluoropyrimidine + platinum compounds	Olaparib + Paclitaxel *n* = 62 Median age years (range): 63.0 (31–77) Males: *n* = 49 (79.0%)	13.1	0.56 (0.35–0.87) *P* = 0.010	3.9	0.80 (80% CI, 0.62–1.03) *P* = 0.131	*n* = 14 Rate = 26.4% (95%CI = NR)	NR	*n* = 46 Rate = 75.4% (95%CI = NR)	NR
		Paclitaxel + Placebo *n* = 62 Median age years (range): 60.5 (25–79) Males: *n* = 44 (71.0%)	8.3		3.6		*n* = 9 Rate = 19.1% (95%CI = NR) Not significant *Evaluable-for-response analysis set		*n* = 46 Rate = 74.2% (95%CI = NR)	
Bang et al. (2017) [47]	Phase III Median follow-up: 11.1 months (Olaparib + Paclitaxel); 9.9 months (Paclitaxel + Placebo) Location: 58 study sites in China, Japan, South Korea, and Taiwan Prior Treatment: Fluoropyrimidine + platinum compounds	Olaparib + Paclitaxel *n* = 263 Median age years (range): 58 (49–67) Males: *n* = 174 (66%)	8.8 (7.4–9.6)	0.79 (97.5% CI, 0.63–1.00); *P* = 0.026 (NS) Statistical significance set at *P* < 0.025.	3.7 (3.7–4.2)	0.84 (97.5% CI, 0.67–1.04) *P* = 0.065	*n* = 44 Rate = 17% (95%CI = NR)	OR = 1·69 (97.5% CI 0·92–3·17) *P* = 0·055	NR	NR
		Paclitaxel + Placebo *n* = 262 Median age years (range): 59 (50–65) Males: *n* = 185 (71%)	6.9 (6.3–7.9)		3.2 (2.2–3.5)		*n* = 28 Rate = 11% (95%CI = NR)		NR	
		ATM low, HER2 negative subgroup: Olaparib + Paclitaxel *n* = 48 Median age years (range): 58 (50–66) Males: *n* = 31 (65%)	12.0 (7.8–18.1)	0.73 (97.5% CI, 0.40–1.34); *P* = 0.25	5.3 (3.5–9.0)	0.74 (97.5% CI, 0.42–1.29); *P* = 0.22	*n* = 12 Rate = 25% (95%CI = NR)	OR = 4·24 (97.5% CI 0·95–23·23) *P* = 0·031	NR	NR
		ATM low, HER2 negative subgroup: Paclitaxel + Placebo *n* = 46 Median age years (range): 60·5 (57–69) Males: *n* = 31 (67%)	10.0 (6.4–13.3)		3.7 (1.9–5.3)		*n* = 11 Rate = 5% (95%CI = NR)		NR	
RAINBOW trial Wilke et al. (2014) [73]	Phase III Median follow-up: 7.9 months Location: 27 countries across North & South America, Europe, Asia, & Australia Prior Treatment: Fluoropyrimidine + platinum compounds	Ramucirumab + Paclitaxel *n* = 330 Median age years (range): 61 (25–83) Males *n* = 229 (69%)	9.6 (8.5–10.8)	0.807 (0.678–0.962) *P* = 0.017	4.4 (4.2–5.3)	0.635 (0.536–0.752) *P* < 0.0001	*n* = 92 Rate = 28.0% (95%CI = 23–33)		NR	NR
		Paclitaxel + Placebo *n* = 335 Median age years (range): 61 (24–84) Males *n* = 243 (73%)	7.4 (6.3–8.4)		2.9 (2.8–3.0)		*n* = 54 Rate = 16.0% (95%CI = 13–20) *P* = 0.0001		NR	
Muro et al. (2016) [46]	Phase III *n* = 223 Median follow-up (Overall RAINBOW trial): 7.9 months Location: Hong Kong, Japan, South Korea, Singapore & Taiwan Prior Treatment: Fluoropyrimidine + platinum compounds	East Asians: Ramucirumab + Paclitaxel *n* = 109 Median age years (range): 62 (28–76) Males *n* = 73 (67%)	12.1	0.986 (0.727–1.337) *P* = 0.929	5.5	0.628 (0.473–0.834)	*n* = 37 Rate = 34.0% (95%CI = NR)	East Asians vs. Non-East Asians OR, 2.24 (1.18–4.24) *P* = 0.0134	*n* = 92 Rate = 85.0% (95%CI = NR)	NR
		East Asians: Paclitaxel + Placebo *n* = 114 Median age years (range): 62 (28–81) Males *n* = 81 (71%)	10.5		2.8		*n* = 23 Rate = 20.0% (95%CI = NR)		*n* = 66 Rate = 59.0% (95%CI = NR)	
	*n* = 442 Location: Hong Kong, Japan, South Korea, Singapore & Taiwan	Non-East Asian cohort: Ramucirumab + Paclitaxel Median age years (range): 60 (25–83) Males *n* = 156 (71%) *n* = 221	8.5	0.732 (0.591–0.907) Treatment-by-region interaction (OS) not significant; *P* = 0.1178	4.2	0.639 (0.518–0.788)	*n* = 55 Rate = 25.0% (95%CI = NR)		*n* = 175 Rate = 80.0% (95%CI = NR)	NR
		Non-East Asian cohort: Ramucirumab + Paclitaxel Median age years (range): 60 (24–84) Males *n* = 162 (73%) *n* = 221	5.9		2.9		*n* = 31 Rate = 14.0% (95%CI = NR)		*n* = 140 Rate = 65.0% (95%CI = NR)	
Shitara et al. (2016) [74]	Phase III Median follow-up (Overall RAINBOW trial): 7.9 months *n* = 140 Median age years (range): 64.0 (29–76) Males *n* = 99 (70.7%)	Japanese cohort: Ramucirumab + Paclitaxel *n* = 68	11.4	0.880 (0.603–1.284); *P* = 0.5113	5.6	0.503 (0.348–0.728) *P* = 0.0002	*n* = 28 Rate = 41.2% (95%CI = NR)	*P* = 0.0035	*n* = 57 Rate = 83.80% (95%CI = NR)	NR
		Japanese cohort: Paclitaxel + Placebo *n* = 72	11.5		2.8		*n* = 14 Rate = 19.4% (95%CI = NR)		*n* = 37 Rate = 52.10% (95%CI = NR)	
	*n* = 398 Median age years (range): 60.0 (24–84) Males n = 287 (72.1%) Location: Australia, Europe, Israel, US Prior Treatment: Fluoropyrimidine + platinum compounds	Western cohort: Ramucirumab + Paclitaxel *n* = 198	8.6	0.7326 (0.580–0.909) *P* = 0.005	4.2	0.631 (0.506–0.786) *P* = 0.0001	*n* = 53 Rate = 26.8% (95%CI = NR)	*P* = 0.0004	*n* = 155 Rate = 79.10% (95%CI = NR)	NR
		Western cohort: Paclitaxel + Placebo *n* = 200	5.9		2.8		*n* = 26 Rate = 13.0% (95%CI = NR)		*n* = 122 Rate = 61.90% (95%CI = NR)	
Satoh et al. (2014) [44]	Phase III Median follow-up: NR Median age years (range): 61 (22–80) Males *n* = 207 (79%) Location: 48 centres in mainland China, Japan, South Korea, and Taiwan Prior Treatment: Fluoropyrimidine + platinum compounds	Lapatinib + Paclitaxel *n* = 132	11.0	0.84 (0.64–1.11) *P* = 0.1044	5.5	0.85 (0.63–1.13) *P*= 0.2441	*n* = 35 Rate = 27.0% (95%CI = 9.2–34.9)	NR OR, 3.85, 95% CI (1.80–8.87) *P* < 0.001	NR	NR
		Paclitaxel *n* = 129	8.9		4.4		*n* = 11 Rate = 9.0% (95%CI = 4.3–14.7)		NR	
CCOG0701. Nakanishi et al. (2016) [53]	Phase II Median follow-up: NR Location: Japan Prior Treatment: Fluoropyrimidine + platinum compounds	Paclitaxel + S-1 *n* = 40 Median age years (range): 64 (42–79) Males *n* = 29 (76%)	10.0 (0.4–74.1)	0.834 (0.511–1.359)	4.6 (0.4–74.1)	0.862 (0.543–1.367) difference NS Unadjusted OS & PFS	n = NR Rate = 22.0% (95%CI = NR)	*P* = 0.767	NR	NR
		Paclitaxel *n* = 49 Median age years (range): 62 (38–80) Males *n* = 34 (85%)	10.0 (1.3–72.0)		4.6 (0.4–59.6)		n = NR Rate = 27.0% (95%CI = NR)		NR	
Paclitaxel versus Other Treatments										
JCOG0407. Nishina et al. (2016) [54]	Phase II Median follow-up: NR Location: Japan Prior Treatment: Fluoropyrimidine alone	Best available Fluorouracil *n* = 49 Median age years (range): 59 (30–74) Males *n* = 33 (NR%)	7.7 (6.7–9.0)	0.89 (0.57–1.38) *P* = 0.298	2.4 (1.7–3.6)	0.58 (0.38–0.88) *P* = 0.005	NR	NR	NR	NR
		Paclitaxel *n* = 51 Median age years (range): 64 (39–75) Males *n* = 36 (NR%)	7.7 (6.0–9.7)		3.7 (2.6–3.7)		NR		NR	
Van Cutsem et al. (2017) [76]	Phase II Median OS follow-up: 4.8 months (AZD4547); 5.1 months (Paclitaxel) Median PFS follow-up: 1.77 months (AZD4547); 2.12 months (Paclitaxel) Mean age years (SD): 61.2 (11.0) Males *n* = 51 (71.8%) Location: 56 centres in Asia, North America, and Europe Prior Treatment: Unspecified prior chemotherapy (one prior line; regimen NR)	AZD4547 *n* = 41	5.5 (95% CI, NR)	1.31 (80% CI, 0.89–1.95) *P* = 0.8156	1.8	1.57 (80% CI, 1.12–2.21) *P* = 0.9581	n = NR Rate = 2.6% (95%CI = NR)	OR, 0.09 (80% CI, 0.02–0.35) *P* = 0.9970	NR	NR
		Paclitaxel *n* = 30	6.6 (95% CI, NR)		3.5		n = NR Rate = 23.3% (95%CI = NR)		NR	
Lee et al. (2019) [61]	Phase III Median follow-up: NR Location: Korea Prior Treatment: Fluoropyrimidine + platinum compounds + trastuzumab (in the HER2-positive setting)	Paclitaxel *n* = 54 Median age years (range): 58.5 (38–82) Males *n* = 38 (70.4%)	8.57 (7.1–10.0)	1.39 (0.91–2.11) *P* = 0.126	3.47 (2.2–4.7)	1.27 (0.86–1.88) *P* = 0.234	*n* = 6 Rate = 15.8% (95%CI = NR)	*P* = 0.355 Assessed in cohort with ≥ one measurable lesion (paclitaxel (*n* = 38/54) irinotecan (*n* = 44/58)	N = NR Rate = 32.70% (95%CI = NR)	*P* = 0.177
		Irinotecan *n* = 58 Median age years (range): 59 (38–77) Males *n* = 40 (69.0%)	7.03 (5.6–8.4)		2.10 (1.4–2.8)		*n* = 6 Rate = 13.6% (95%CI = NR)		n = NR Rate = 45.60% (95%CI = NR)	
Hironaka et al. (2013)[49]	Phase III Median follow-up: NR Location: Japan Prior Treatment: Fluoropyrimidine + platinum compounds	Paclitaxel *n* = 108 Median age years (range): 64.5 (37–75) Males *n* = 84 (77.7%)	9.5 (8.4–10.7)	1.13 (0.86–1.49) *P* = 0.38	3.6 (3.3–3.8)	1.14 (0.88–1.49) *P* = 0.33	*N* = 19 Rate = 20.9% (95%CI = NR)	NR Assessed in cohort with ≥ one measurable lesion at baseline.	NR	NR
		Irinotecan *n* = 111 Median age years (range):65 (38–75) Males *n* = 87 (78.4%)	8.4 (7.6–9.8)		2.3 (2.2–3.1)		*n* = 12 Rate = 13.6% (95%CI = NR) *P* = 0.24			
Kawase et al. (2021) [41]	Phase II Median follow-up: 11.3 months Median age years (range): 65 (31–74) Location: Japan Prior Treatment: Fluoropyrimidine + platinum compounds	Irinotecan or S-1 + irinotecan *n* = 64		0.979 (0.679–1.412) Log-rank *P* = 0.914	3.6	0.674 (0.468–0.972) Log-rank *P* = 0.034	n = NR Rate = 6.3% (1.7–15.2)		NR	
		Paclitaxel or S-1 + Paclitaxel *n* = 63			4.1		n = NR Rate = 12.7% (5.6–23.5)			
		Irinotecan or Paclitaxel *n* = 85		0.954 (0.644–1.412) Log-rank *P* = 0.814	3.7	1.03 (0.7–1.508.7.508) Log-rank *P* = 0.893	n = NR Rate = 11.8% (5.8–20.6)			
		S-1 + Irinotecan or S-1 + Paclitaxel *n* = 42			3.6		n = NR Rate = 4.8% (0.6–16.2)			
KEYNOTE-063 Chung et al. (2022)[40]	Phase III Open Median follow-up: 24 months Location: Asia Prior Treatment: Fluoropyrimidine + platinum compounds + trastuzumab (in the HER2-positive setting)	Pembrolizumab *n* = 47 Median age years (range): 61 (32–75) Males *n* = 32 (68%)	8 (4–10)	0.99 (0.63–1.54)	2(1–3)	1.62 (1.04–2.52)	*n* = 6 Rate = 13% (95%CI = NR)	NR	*n* = 5 Rate = 11% (95%CI = NR)	NR
		Paclitaxel *n* = 47 Median age years (range): 61 (37–91) Males *n* = 37 (79%)	8 (5–11)		4(3–6)		*n* = 9 Rate = 19% 95%CI = NR)		*n* = 28 Rate = 64% (95%CI = NR)	
KEYNOTE 061. Fuchs et al. (2022)[69]	Phase III Median follow-up: 4 years & 4 months Location: Global *n* = 592 Prior Treatment: Fluoropyrimidine + platinum compounds	Pembrolizumab *n* = 296 Median age years (range): 62.5 (27–87) Male *n* = 202 (68.2%)	9.1 (6.2–10.7)	0.81 (0.66–1.00.66.00)	1.5 (1.4–2.0.4.0)	1.25(1.02–1.54)	*n* = 32 Rate = 16.3% (95% CI) = NR)	NR	*n* = 44 Rate = 15% (95% CI = NR)	NR
		Paclitaxel *n* = 296 Median age years (range): 60.0 (20–86) Male *n* = 208 (70.3%)	8.3 (7.6–9.0.6.0)		4.1 (3.2–4.3)		*n* = 27 Rate = 13.6% (95%CI = NR)		*n* = 97 Rate = 35% (95%CI = NR)	
KEYNOTE-061. Shitara et al. (2018) [77]		PD-L1 CPS ≥ 1 subgroup: Pembrolizumab *n* = 196 Median age years (range): 64·0 (57–70·5) Male *n* = 146 (74%)	9.1 (6.2–10.7)	HR, 0.82 (0.66–1.03); *P* =0.0421	1.5 (1.4–2.0)	HR, 1.27 (1.03–1.57)	*n* = 31 Rate = 16% (95%CI = NR)	NR	*n* = 42 Rate = 14% (95%CI = NR)	NR
	Location: Global Prior Treatment: Fluoropyrimidine + platinum compounds + trastuzumab (in the HER2-positive setting)	PD-L1 CPS ≥ 1 subgroup: Paclitaxel *n* = 199 Median age years (range): 61·0 (54–68) Male *n* = 140 (70%)	8.3 (7.6–9.0)		4.1 (3.1–4.2)		*n* = 27 Rate = 14% (95%CI = NR)		*n* = 96 Rate = 35% (95%CI = NR) Assessed in *n* = 570 who received ≥ one dose of study treatment, irrespective of PD-L1 CPS.	
Kang et al. (2018) [62]	Phase III Median follow-up: NR Median age years (range): 59 (27–83) Male *n* = 185 (78.4) Location: Korea Prior Treatment: Fluoropyrimidine alone or Fluoropyrimidine + platinum compounds	DHP107 (oral formulation of Paclitaxel) *n* = 118	9.7 (7.1–11.5)	1.04 (0.76–1.41) *P* = 0.824	3.0 (1.7–4.0)	0.85 (0.64–1.13)	NR	NR	NR	NR
		Paclitaxel *n* = 118	8.9 (7.1–12.2)		2.6 (1.8–2.8)		NR		NR	
Shitara et al. (2017)[55]	Phase III Median follow-up (OS): 9.99 months Location: Japan Prior Treatment: Fluoropyrimidine + platinum compounds	Nab-Paclitaxel once every three weeks *n* = 243	5.6 (4.4–6.7)	versus Paclitaxel: 1.06 (95% CI 0.87–1.31); *P* = 0.062	3.8 (3.5–4.4)	versus Paclitaxel: 1.03 (0.85–1.24) *P* = 0.778	n = NR Rate = 25% (95%CI = NR)	versus Paclitaxel: (18.6–33.1) *P* = 0.897	NR	NR
		Nab-Paclitaxel once every week *n* = 240	6.9 (2.1–11.7)	versus Paclitaxel: 0.97 (97.5% CI, 0.76–1.23); *P* = 0.0085	5.3 (4.0–5.6)	versus Paclitaxel: 0.88 (0.73–1.06) *P* = 0.176	n = NR Rate = 33% (95%CI = NR)	versus Paclitaxel: (25.2–40.8) *P* = 0.106	NR	NR
		Paclitaxel *n* = 243	10.3 (8.7–11.4)		3.8 (3.7–3.9)		n = NR Rate = 24% (95%CI = NR)	(18.0–31.4)	NR	NR
Docetaxel versus Other Treatments										
COUGAR-02 Ford et al. (2014) [68]	Phase III Median follow-up: 12 months Location: 30 centres in UK Prior Treatment: Fluoropyrimidine + platinum compounds	Docetaxel *n* = 84 Median age years (range): 65 (28–84) Male *n* = 69 (82%)	5.2 (4.1–5.9)	0.67 (0.49–0.92) *P* = 0.01	NR	NR	NR	NR	NR	NR
		Active symptom control *n* = 84 Median age years (range): 66 (36–84) Male *n* = 67 (80%)	3.6 (3.3–4.4)				NR			
Yi et al. (2012) [42]	Phase II Median follow-up: NR Location: Single centre study in Korea Prior Treatment: Fluoropyrimidine + platinum compounds	Docetaxel + sunitinib *n* = 56 Median age years (range): 54.0 (20–72) Male *n* = 40 (71.4)	8.0 (5.4–10.6)	0.94 (0.60–1.49) *P* = 0.802	NR	NR	*n* = 23 Rate = 41.1% (95%CI = NR)	*P* = 0.002	*n* = 26 Rate = 46.4% (95%CI = NR)	*P* = 0.112
		Docetaxel *n* = 49 Median age years (range): 52 (36–70) Male *n* = 33 (67.3%)	6.6 (3.6–9.7)		NR		*n* = 7 Rate = 14.3% (95%CI = NR)		*n* = 15 Rate = 30.60% (95%CI = NR)	
Kim et al. (2015) [59]	Phase II Median follow-up: NR Location: Korea Prior Treatment: Fluoropyrimidine + platinum compounds	Docetaxel *n* = 27 Median age years (range): 54 (NR) Male *n* = 24 (NR%)	7.2 (6.0–8.4)	*P* = 0.353	2.0 (1.2–2.9)	*P* = 0.002	n = NR Rate = 14.8% (95%CI = NR)	*P* = 0.40 NR	NR	NR
		Docetaxel + Oxaliplatin *n* = 25 Median age years (range): 59 (NR) Male *n* = 25 (NR%)	8.1 (7.6–8.6)		4.9 (3.6–6.6)		n = NR Rate = 24% (95%CI = NR)		NR	
Lee et al. (2017) [60]	Phase II Median follow-up: 7.3 months Location: Korea Prior Treatment: Fluoropyrimidine + platinum compounds	Docetaxel *n* = 23 Median age years (range): 56 (34–68) Male *n* = 18 (78.3%)	10.0 (7.8–12.2)		1.3 (1.0–1.5)		*n* = 1 Rate = 4.3% (95%CI = NR)		*n* = 10 Rate = 43.5% (95%CI = NR)	
		Docetaxel + Cisplatin *n* = 23 Median age years (range): 55 (38–74) Male *n* = 20 (87.0%)	5.6 (4.4–6.7)	Docetaxel + Cisplatin versus Docetaxel: *P* = 0.035	1.8 (0.8–2.9)	Docetaxel + Cisplatin versus Docetaxel: : *P* = 0.804	*n* = 1 Rate = 4.3% (95%CI = NR)	Docetaxel + Cisplatin versus Docetaxel: *P* > 0.990	*n* = 15 Rate = 62.5% (95%CI = NR)	Docetaxel + Cisplatin versus Docetaxel: *P* = 0.155
		Docetaxel + S-1 *n* = 23 Median age years (range): 55 (39–68) Male *n* = 14 (60.9%)	6.9 (2.1–11.7)	Docetaxel + S-1 versus Docetaxel : *P* = 0.421	2.7 (1.0–4.4.0.4)	Docetaxel + S-1 versus Docetaxel: *P* = 0.034	*n* = 2 Rate = 8.7% (95%CI = NR)	Docetaxel + S-1 versus Docetaxel : *P* > 0.990	*n* = 8 Rate = 32.9% (95%CI = NR)	Docetaxel + S-1 versus Docetaxel *P* = 0.382
GATSBY Thuss-Patience et al. (2017) [75]	Phase II & Phase III Median follow-up: 15.4 months (Taxane); 17.5 months (Trastuzumab emtansine) Location: Globally Prior Treatment: Fluoropyrimidine + platinum compounds	Taxane *n* = 117 Median age years (range): 62·0 (27–80) Male *n* = 95 (81%)	8.6 (7.1–11.2)	1.15 (0.87–1.51) *P* = 0.86	2.9 (2.8–4.0)	1.13 (0.89–1.43) *P* = 0.31	*n* = 20 Rate = 19.6% (95%CI = NR)	*P* = 0.8406	*n* = 78 Rate = 70% (95%CI = NR)	NR
		Trastuzumab emtansine *n* = 228 Median age years (range): 62·0 (19–79) Male *n* = 177 (78%)	7.9 (6.7–9.5)		2.7 (1.6–2.7)		*n* = 42 Rate = 20.6% (95%CI = NR)		*n* = 134 Rate = 60% (95%CI = NR)	
Irinotecan versus Other Treatments										
Higuchi et al. (2014) [50]	Phase III Median follow-up: NR Location: Japan Prior Treatment: Fluoropyrimidine + platinum compounds	Irinotecan + Cisplatin *n* = 64 Median age years (range): 66 (29–80) Males n = 49 (77%)	10.7	1.00 (0.69–1.44) *P* = 0.9823	3.8	0.68 (0.47–0.98) *P* = 0.0398	*n* = 14 Rate = 22.0% (95%CI = 13–34)	*P* = 0.4975	NR	NR
		Irinotecan *n* = 63 Median age years (range): 67 (49–78) Males *n* = 55 (87%)	10.1		2.8		*n* = 10 Rate = 16.0% (95%CI = 8–27)			
Nishikawa et al. (2015) [52]	Phase III Median follow-up: 59 months Location: Japan Prior Treatment: Fluoropyrimidine alone	Irinotecan + Cisplatin *n* = 84 Median age years (range): 67 (36–85) Males *n* = 68 (81%)	13.9 (10.8–17.6)	0.834 (0.596–1.167) *P* = 0.288	4.6 (3.4–5.9)	0.860 (0.610–1.203) *P* = 0.376	NR	NR	NR	NR
		Irinotecan *n* = 84 Median age years (range): 68 (35–87) Males *n* = 63 (75%)	12.7 (10.3–17.2)		4.1 (3.3–4.9)		NR		NR	
Thuss-Patience et al. (2011) [65]	Phase III Median follow-up: NR Location: Germany Prior Treatment: Fluoropyrimidine + platinum compounds or anthracycline or docetaxel.	Irinotecan *n* = 21 Median age years (range): 58 (43–73) Males *n* = 18 (86%)	4.0 (3.6–7.5)	0.48 (0.25–0.92) *P* = 0.012	ITT: 2.5 (1.6–3.9) Per-Protocol: 2.6 (1.7–4.3) NR (Results provided for Irinotecan arm only)	NR	NR	NR	NR	NR
		Best Supportive Care *n* = 19 Median age years (range): 55 (35–72) Males *n* = 11 (58%)	2.4 (1.7–4.9)				NR			
Sym et al. (2013) [43]	Phase II Median follow-up: NR Location: Korea Prior Treatment: Fluoropyrimidine + platinum compounds	Irinotecan *n* = 29 Median age years (range): 60 (45–76) Males *n* = 20 (69%)	5.8 (3.0–8.7)	1.21 (0.69–2.11) *P* =0.514	2.2 (0.2–4.3)	1.20 (0.72–2.02) *P* = 0.481	*n* = 5 Rate = 17.2% (95%CI = 3.4–30.9)	*P* = 0.525	*n* = 21 Rate = NR (95%CI = NR)	*P* = 0.067
		FOLFIRI *n* = 30 Median age years (range): 61 (30–75) Males *n* = 14 (47%)	6.7 (5.3–8.2)		3.0 (2.0–3.7)		*n* = 6 Rate = 20.0% (95%CI = 5.6–34.3)		*n* = 28 Rate = NR (95%CI = NR)	
Roy et al. (2013) [71]	Phase II Median follow-up: NR Location: UK, Spain, Taiwan, Croatia, Korea and Bosnia Prior Treatment: Unspecified prior chemotherapy (one prior line; regimen NR)	PEP02 (liposomal irinotecan) *n* = 44 Median age years (range): 56 (38–81) Males *n* = 35 (79.5%)	7.3 (3.84–9.17)	NR	2.7 (1.54–3.65)	NR	*n* = 6 Rate = 13.6% (95%CI = NR)	NR	*n* = 41 Rate = 38.6% (95%CI = NR)	NR
		Irinotecan *n* = 44 Median age years (range): 62(33–79) Males *n* = 34 (77.3%)	7.8 (4.90–9.20)		2.6 (1.48–4.34)		*n* = 3 Rate = 6.8% (95%CI = NR)		*n* = 43 Rate = 34.1% (95%CI = NR)	
		Docetaxel *n* = 44 Median age years (range): 58(33–81) Males *n* = 34 (77.3%)	7.7 (5.32–12.32)		2.7 (1.41–5.45)		*n* = 7 Rate = 15.9% (95%CI = NR)		*n* = 40 Rate = 15.9% (95%CI = NR)	
Tanabe et al. (2015) [51]	Phase II & Phase III Median follow-up: 9.07 months	S-1 + Irinotecan *n* = 145 Median age years (range): 67 (37–84) Males (*n* = 99 (68%)	8.8 (IQR, 5.6–15.7)	0.99 (0.78–1.25) *P* = 0.92	3.8 (IQR, 1.9–6.6)	0.85 (0.67–1.07) *P* = 0.16	*n* = 9/118 Rate = 7.6% (95%CI = 2.8–12.4) Measurable lesions: S-1 + Irinotecan (*n* = 118); Irinotecan (*n* = 122)	NR	NR	NR
	Location: Japan Prior Treatment: Fluoropyrimidine + platinum compounds	Irinotecan *n* = 148 Median age years (range): 66 (22–83) Males *n* = 109 (74%)	9.5 (IQR, 5.6–14.1)		3.4 (IQR, 1.6–5.3)		*n* = 9/122 Rate = 7.4% (95%CI = 2.7–12.0) NS		NR	NR
Satoh et al. (2015) [45]	Phase II Median follow-up: 242.5 days Median age years (range): 61.5 (27–75) Males *n* = 66 (80.5%) Location: Japan & Korea Prior Treatment: Fluoropyrimidine + platinum compounds	Nimotuzumab + Irinotecan *n* = 40 Median age years (range): 60.0 (27–75) Males *n* = 33 (82.5%)	250.5 days (171.0–306.0)	0.994 (0.618–1.599) *P* = 0.9778	73.0 days (55.0–112.0)	0.860 (0.516–1.435) *P* = 0.5668	n = NR Rate = 18.4% (95%CI = NR)	NR	n = NR Rate = 77.5% (95%CI = NR)	NR
		Irinotecan *n* = 42 Median age years (range): 63.5 (32–75) Males *n* = 33 (78.6%)	232.0 days (148.0–319.0)		85.0 days (37.0–93.0)		n = NR Rate = 10.3% (95%CI = NR) *P* = 0.3060		n = NR Rate = 64.3% (95%CI = NR)	NR
Targeted Treatments & Immunotherapy Treatments										
Fuchs et al. (2014) [72]	Phase III Median follow-up: NR Location: Globally Prior Treatment: Fluoropyrimidine + platinum compounds	Ramucirumab *n* = 238 Median age years (range): 60 (52–67) Males n = 169 (71%)	5.2 (IQR, 2.3–9.9)	0.776 (0.603–0.998) *P* = 0.047	2.1 (IQR, 1.3–4.2)	0.483 (0.376–0.620) *P* < 0.0001	*n* = 7 Rate = 3.0% (95%CI = NR)	NR	NR	NR
		Placebo *n* = 117 Median age years (range): 60 (51–71) Males *n* = 79 (68%)	3.8 (IQR, 1.7–7.1)		1.3 (IQR, 1.1–2.1)		*n* = 3 Rate = 3.0% (95%CI = NR) *P* = 0.76		NR	
Tougeron et al. (2024) [81]	Phase II Median follow-up: 20.3 months (FOLFIRI + durvalumab); 23.2 months (FOLFIRI + durvalumab + tremelimumab) Median age years (range): 59.7 (24.7–83.3) Males: *n* = 64 (69.6%) Location: France Prior Treatment: Fluoropyrimidine + platinum compounds	FOLFIRI + durvalumab *n* = 46	13.2 (6.6–15.6)	NR	3.8 (3.0–7.4.0.4)	NR	*N* = 16 Rate = 34.7% (95%CI = NR)	NR	*n* = 22 Rate = 47.8% (95%CI = NR)	NR
		FOLFIRI + durvalumab + tremelimumab *n* = 46	9.5 (7.1–11.3)		5.4 (2.9–6.4)		*N* = 17 Rate = 37.7% (95%CI = NR)		*N* = 22 Rate = 47.8% (95%CI = NR)	
Lorenzen et al. (2022) [64]	Phase II Median follow-up (OS): 6.8 months Median age years (range) 61 (NR) Male *n* = 74 (67%) Location: Germany Prior Treatment: Fluoropyrimidine + platinum compounds or anthracycline or docetaxel	FOLFIRI + Ramucirumab *n* = 72	6.8 (5.1–11.1)	0.97 (0.62–1.52)	3.9 (2.8–6.8)	0.73 (0.48–1.11)	*n* = 16 Rate = 22% (95%CI = NR)	NR	*n* = 54 Rate = 75% (95%CI = NR)	NR
		Paclitaxel + Ramucirumab *n* = 38	7.6 (6.1–11.5)		3.7 (2.1–5.5)		*n* = 4 Rate = 11% (95%CI = NR)		*n* = 23 Rate = 68% (95%CI = NR) Safety Population: FOLFIRI + Ramucirumab (*n* = 72) Paclitaxel + Ramucirumab (*n* = 34)	
Yan et al. (2022) [82]	Phase NR Median follow-up: NR Median age years (range): NR Location: China Prior Treatment: Fluoropyrimidine + platinum compounds	Intermittent -Apatinib + Docetaxel *n* = 38 Males n = 25 (65.8%)	9.00 (5.31–12.70)	NR	3.88 (1.72–6.03)	NR	*n* = 8 Rate = 21.1% (95%CI = NR)	NR	n = NR Rate = 36.8% (95%CI = NR)	NR
		Continuous – Apatinib + Docetaxel *n* = 38 Males n = 28 (73.7%)	9.40 (5.20–13.59) *P* = 0.310		3.98 (1.06–6.90) *P* = 0.546		*n* = 7 Rate = 18.4% (95%CI = NR) *P* = 0.773		n = NR Rate = 29.5% (95%CI = NR)	
Wei et al. (2024) [83]	Phase II Median follow-up: 16.2 months Location: China Prior Treatment: Fluoropyrimidine + platinum compounds + trastuzumab (in the HER2-positive setting)	Apatinib + Toripalimab *n* = 25 Median age years (range): 59.2 (27–75)	8.3	1.221; (0.641–2.328) *P* = 0.539	2.77	0.885 (0.502–1.560) *P* = 0.660	*n* = 5 Rate = 20.0% (95%CI = NR)	NR	*n* = 6 Rate = 24.0% (95%CI = NR)	NR
		Irinotecan or Paclitaxel or Docetaxel *n* = 26 Median age years (range): 59.8 (27–77)	9.8		2.33		*n* = 6 Rate = 23.0% (95%CI = NR) *P* = 0.368		*n* = 9 Rate = 36.0% (95%CI = NR)	
**Second- & Later-Line Setting (n = 5)**										
Lorenzen et al. (2022) [66]	Phase III Median follow-up: 6.2 months (Paclitaxel + Everolimus); 5.6 months (Paclitaxel) *n* = 300 Median age years (range): 62 (29–86) Male *n* = 231 (77%) Location: 50 centres in Germany Prior Treatment: Fluoropyrimidine + platinum compounds	Paclitaxel + Everolimus *n* = 150	6.1 (4.2–6.6)	0.93 (0.73–1.18) *P* = 0.544	2.2 (2.1–2.7)	0.88 (0.70–1.11) *P* = 0.273	n = NR/143 Rate = 8.0% (95%CI = 4.2–13.6)	NR	n = NR/143 Rate = 78.3% (95%CI = NR)	NR
		Paclitaxel *n* = 150	5.0 (4.4–6.4		2.1 (1.9–2.5		n = NR/147 Rate = 7.3% (95%CI = 3.7–12.7)		n = NR/147 Rate = 69.4% (95%CI = NR)	
Kang et al. (2012) [63]	Phase III Median follow-up: 20 months *n* = 202 Median age years (range): 56 (31–83) Male *n* = 137 (68%) Location: Korea Prior Treatment: Fluoropyrimidine + platinum compounds	Docetaxel or Irinotecan *n* = 133	5.3 (4.1–6.5)	0.657 (0.485–0.891) *P* = 0.007	NR	NR	NR	NR	NR	NR
		Best Supportive Care *n* = 69	3.8 (3.1–4.5)		NR		NR		NR	
Shitara et al. (2014) [56]	Phase II Median follow-up: NR Location: Japan Prior Treatment: Fluoropyrimidine + platinum compounds	Dose-escalated Paclitaxel *n* = 44 Median age years (range): 62 (29–78) Male *n* = 32 (73%)	11.8 (7.6–16.3)	0.75 (0.45–1.22) *P* = 0.12	4.3 (3.0–5.7)	0.55 (0.34–0.90) *P* = 0.017	n = NR Rate = 30.3% (95%CI = 15.6–48.7)	NR *P* = 0.2	NR	NR
		Paclitaxel *n* = 45 Median age years (range): 65 (33–80) Male *n* = 29 (64%)	9.6 (7.4–11.7)		2.5 (1.8–3.7)		n = NR Rate = 17.1% (95%CI = 6.6–33.7)		NR	
Fushida et al. (2016) [57]	Phase II Median follow-up: NR Location: Japan Prior Treatment: Fluoropyrimidine + platinum compounds	Paclitaxel (*n* = 33) Median age years (range): 68 (49–84) Male n = NR (NR%)	9.8	1.19 (0.702–2.026) *P* = 0.51	4.5	1.29 (0.753–2.211) *P* = 0.35	NR	NR	NR	NR
		Paclitaxel + Valproic acid (*n* = 31) Median age years (range): 67 (31–83) Male n = NR (NR%)	8.7		3.0		NR		NR	
Moehler et al. (2016) [67]	Phase II Median follow-up: NR Location: Germany Prior Treatment: Fluoropyrimidine + platinum compounds	Na-FOLFIRI + sunitinib *n* = 45 Median age years (range): 62 (37–76) Male *n* = 33 (73%)	10.4 (4.5–10.9)	0.82 (0.50–1.34) *P* = 0.42	3.5 (1.4–5.6)	1.11 (0.70–1.74) *P* = 0.66	*n* = 9 Rate = 20.0% (95%CI = NR)	NR	NR	NR
		Na-FOLFIRI + Placebo *n* = 45 Median age years (range): 57 (28–84) Male *n* = 30 (67%)	8.9 (5.9–11.8)		3.3 (1.5–5.2)		*n* = 13 Rate = 29.0% (95%CI = NR)		NR	
**Third- and Later-line Setting (n = 2)**										
Shitara et al. (2020) [79]	Phase II Median follow-up: NR Location: Japan, South Korea Prior Treatment: Fluoropyrimidine + platinum compounds + trastuzumab (in the HER2-positive setting)	Trastuzumab deruxtecan *n* = 125 Median age years (range) 65 (34–82)	12.5 (9.6–14.3)	0.59 (0.39–0.88) *P* = 0.01	5.6 (4.3–6.9)	0.47 (0.31–0.71)	*n* = 61/119 Rate = 51.0% (95%CI = NR)	NR	*n* = 107 Rate = 85.6% (95%CI = NR)	NR
		*n* = 62 (Irinotecan *n* = 55); Paclitaxel *n* = 7) Median age years (range) 66 (28–82)	8.4 (6.9–10.7)		3.5 (2.0- 4.3)		*n* = 8/56 Rate = 14.0% (95%CI = NR)		*n* = 35 Rate = 56.5% (95%CI = NR)	
Shitara et al. (2018) [78]	Phase III Median follow-up: 10.7 months Location: Global Prior Treatment: Fluoropyrimidine + platinum compounds or taxane-based chemotherapy	Trifluridine-tipiracil *n* = 337 Median age years (range) 64 (56–70) Male *n* = 252 (75%)	5.7 (4.8–6.2)	0.69 (0.56–0.85) *P* = 0.00029	2.0 (1.9–2.3)	0.57 (0.47–0.70) *P* < 0.0001	*n* = 13/290 Rate = 4.0% (95%CI = 2–8)	*P* = 0.28	*n* = 267 Rate = 80.0% (95%CI = NR)	NR
		Placebo + Best supportive care *n* = 170 Median age years (range) 63 (56–69) Male *n* = 117 (69%)	3.6 (3.1–4.1)		1.8 (1.7–1.9)		*n* = 3/145 Rate = 2.0% (95%CI = < 1–6)		*n* = 97 Rate = 58.0% (95%CI = NR)	

CI = Confidence Interval; NR = Not reported; NS: Not Significant; OR = odds ratio

An RLR Protocol was developed and registered with PROSPERO (PROSPERO 2024 CRD42024568042). The RLR was conducted in line with RLR Approach 1 as described by Tricco et al. [25] and Smela et al. [26]. The modified PRISMA-NMA Reporting Checklist, as outlined by National Institute for Health and Care Excellence (NICE) [27], was followed. This modified checklist contains the subset of items, from the PRISMA Reporting Checklist [28], that are specifically applicable to the reporting of an NMA. ‘The modified PRISMA-NMA Checklist is presented in Appendix (Table 3)’. The protocol was developed to capture data published subsequent to the dissemination by Catenacci et al.

#### Step Two

All citations housed within the SLR dissemination by Catenacci et al. were reviewed to identify eligible RCTs (published 2009 to 19 November 2019 inclusive) that had investigated the efficacy and/or safety of the treatments of interest here. Those same inclusion and exclusion criteria and restrictions, described for the RLR, were applied when identifying the relevant RCTs from Catenacci et al.

### Data Extraction

The data extraction and quality assessment processes were applied to all RCTs identified via the RLR and also via the Catenacci et al. dissemination [23]. In line with RLR Approach 1 methodology, one reviewer (SS) independently performed all title and abstract screening, full-text screening, and data extraction. Verifications were conducted by a second reviewer (LM). Two reviewers (DM and NC) also verified the accuracy and comprehensiveness of all data extracted with any disagreements resolved through discussion. After the initial screening, the references of primary studies were scanned to ascertain additional relevant RCTs. Forward citation searching was performed on the identified RCTs. In cases where RCT results were reported in multiple publications, data was extracted from the most comprehensive and/or recent dissemination only.

Templates for data extraction, developed in Microsoft Excel^®^, included the study title, author, year of publication, jurisdiction, key population characteristics, number of trial participants, number of participants in each arm, prior treatments received, and outcomes of interest. The primary outcomes of interest were overall survival (OS), progression-free survival (PFS), while additional outcomes were, objective response rate (ORR) and Grade ≥ 3 treatment-related adverse events (TRAEs). For the time-to-event outcomes (i.e. OS and PFS), hazard ratios (HRs) with 95% confidence intervals (95% CI) were extracted. For dichotomous outcomes (i.e. ORR and Grade ≥ 3 TRAEs), absolute number of events, event rates (with 95% CIs) and relative risk (95% CI) were extracted, where available. The RCTs were categorised according to the line(s) of treatment under investigation (i.e. second-line or second- and later-line, or third- and later-line). For any study where the inclusion or data quality was unclear, discussions ensued, and clarifications were reached with one or two additional reviewers as necessary (DM, NC).

Extracted data was presented in tabular form (see Table 1) and also in a narrative summary.

### Assessment of Risk of Bias

The risk of bias (RoB), associated with each RCT, was evaluated using the Cochrane Collaboration RoB 1 Tool for RCTs (Higgins, Altman et al., 2011). We have followed the lead of several recent Cochrane reviews and other authors who have opted for use of the Cochrane RoB 1 Tool in preference to the Cochrane RoB 2 Tool. These authors have cited implementation and pragmatic constraints associated with the RoB 1 Tool [29–31]. Also, the Cochrane Handbook recognises that use of the RoB 1 Tool remains acceptable given ongoing implementation challenges associated with the RoB 2 Tool [32].This evaluation was performed by one reviewer (SS) with clarifications sought from other reviewers when required. The RoB 1 Tool assesses the RoB, in RCTs, across seven domains: Random Sequence Generation, Allocation Concealment, Blinding of Participants and Personnel, Blinding of Outcome Assessment, Attrition Bias, Reporting Bias and Other Bias. Each domain is evaluated for RoB and determined to be low, unclear, or high.

### Data Synthesis and Analysis

#### Construction of Evidence Networks

The NMA approach taken here was aligned with the European Methodological Guidelines for Quantitative Evidence Synthesis for Direct and Indirect Comparisons [33]. Specifically, evidence synthesis was carried out using Bayesian NMA in the generalised linear modelling (GLM) framework described by Dias et al. [34].

Evidence networks for the outcomes of interest (OS, PFS, ORR and Grade ≥ 3 TRAEs) were constructed.

All RCTs identified via the RLR and via Catenacci et al. [23] were assessed for inclusion in the evidence networks. Where two or more citations reported different data cuts of the same study, the most recent data cut was included. Studies reporting post-hoc subgroup analyses from already-included RCTs were excluded.

Different doses, dosing schedules, or modes of administration of the same treatment of interest were classified as a single node in the evidence networks. Therefore, any RCTs that exclusively compared two such interventions were excluded. Where the intervention in a trial arm consisted of different treatments or combinations of different treatments, these were considered as distinct nodes in the networks. Each outcome-specific evidence network was constructed to include all relevant evidence which informed comparative efficacy or safety of the treatments listed in the PICO. Thus, each evidence network included all RCTs that contributed to a path connecting two or more treatments of interest, while any RCTs that did not lie on such a path were excluded.

Feasibility of evidence synthesis, within each evidence network, relies on the underlying assumption of exchangeability, namely, that if individuals in one trial were substituted into another, the treatment effect observed would be expected to be the same [33, 34]. Exchangeability requires that studies included in the networks be sufficiently similar in terms of patient and study characteristics, to ensure that no effect-modification is present. Thus, characteristics of RCTs were assessed and compared, with particular reference to inclusion and exclusion criteria, the role of biomarker-directed therapy, and prior treatments received.

Any RCTs which differed substantially from the main study pool in terms of these characteristics were excluded from the final evidence networks. In particular, RCTs conducted in different lines of treatment were not to be pooled within the same evidence network. Therefore, the feasibility of developing separate evidence networks in the second-line setting, the second- or later-line setting, and the third- or later-line setting was investigated.

### Statistical Methods

Relative treatment effects for all pairwise comparisons in the final NMAs were estimated, measured as HRs for OS and PFS, and as relative risks (RRs) for ORR, and Grade ≥ 3 TRAEs. For the outcomes of OS and PFS, treatment effects were synthesised on the log-hazard ratio scale using a GLM with a normal likelihood and an identity link function. For the dichotomous outcomes of ORR and Grade ≥ 3 TRAEs, a GLM with a binomial likelihood and a log link function [35] was used. Non-informative prior distributions (specifically, normal distributions with mean 0 and variance 10^6) were specified for all treatment-effect parameters.

Random-effects models were used in the base case to account for the presence of between-study heterogeneity. As the estimation of random-effects models can be problematic in networks with few studies, weakly informative prior distributions were used for the between-study heterogeneity parameters, specifically, a half-normal distribution with standard deviation 0.5. This choice of distribution gives a 95% prior probability that the effect observed in any given study will differ from the mean effect by a factor of less than 3 on the RR or HR scale, and was chosen to reflect the typical extent of heterogeneity encountered in meta-analyses of drugs [36, 37]. Treatment effects were summarised using posterior medians and 95% credible intervals (95%CrI) for the estimated HRs (OS and PFS) and RRs (ORR and Grade ≥ 3 TRAEs).

The relative effectiveness and associated uncertainty for all possible pairs of treatments of interest were summarized in grid tables. The grid tables were developed where the treatment and comparator (for each pair-wise comparison) were named on the X-axis and Y-axis respectively. Relative-treatment effect point estimates, for each pair-wise comparison are described in coloured grid cells. Green indicates a point estimate that favours the treatment; red indicates a point estimate that favours the comparator. The colour intensity indicates the magnitude of the point estimate. The colour intensity however does not indicate statistical significance or the width of the 95%CrI).

Due to the small number of RCTs in the final evidence networks, quantitative assessment of heterogeneity was not possible. Similarly, quantitative analysis of inconsistency could not be carried out due to the absence of closed loops. Therefore, assessment of the underlying assumption of exchangeability was carried out via a qualitative assessment of study and population characteristics.

Analysis was carried out in R (version 4.4.2) and JAGS (version 4.3.1) using the package BUGSnet [38]. The Markov chain Monte Carlo sampling was conducted in JAGS using 3 chains with a total 15,000 iterations per chain, of which 4000 were burn-in iterations. An adaptive phase consisting of 1,000 iterations was also conducted to optimise the sampler. No thinning of samples was carried out. Model convergence was assessed via examination of trace plots, posterior density plots of model parameters, and Gelman-Rubin and Geweke convergence diagnostics as described by Du et al. [39].

## Results

### Search Strategy for Eligible Randomised Controlled Trials

**Fig. 1:**
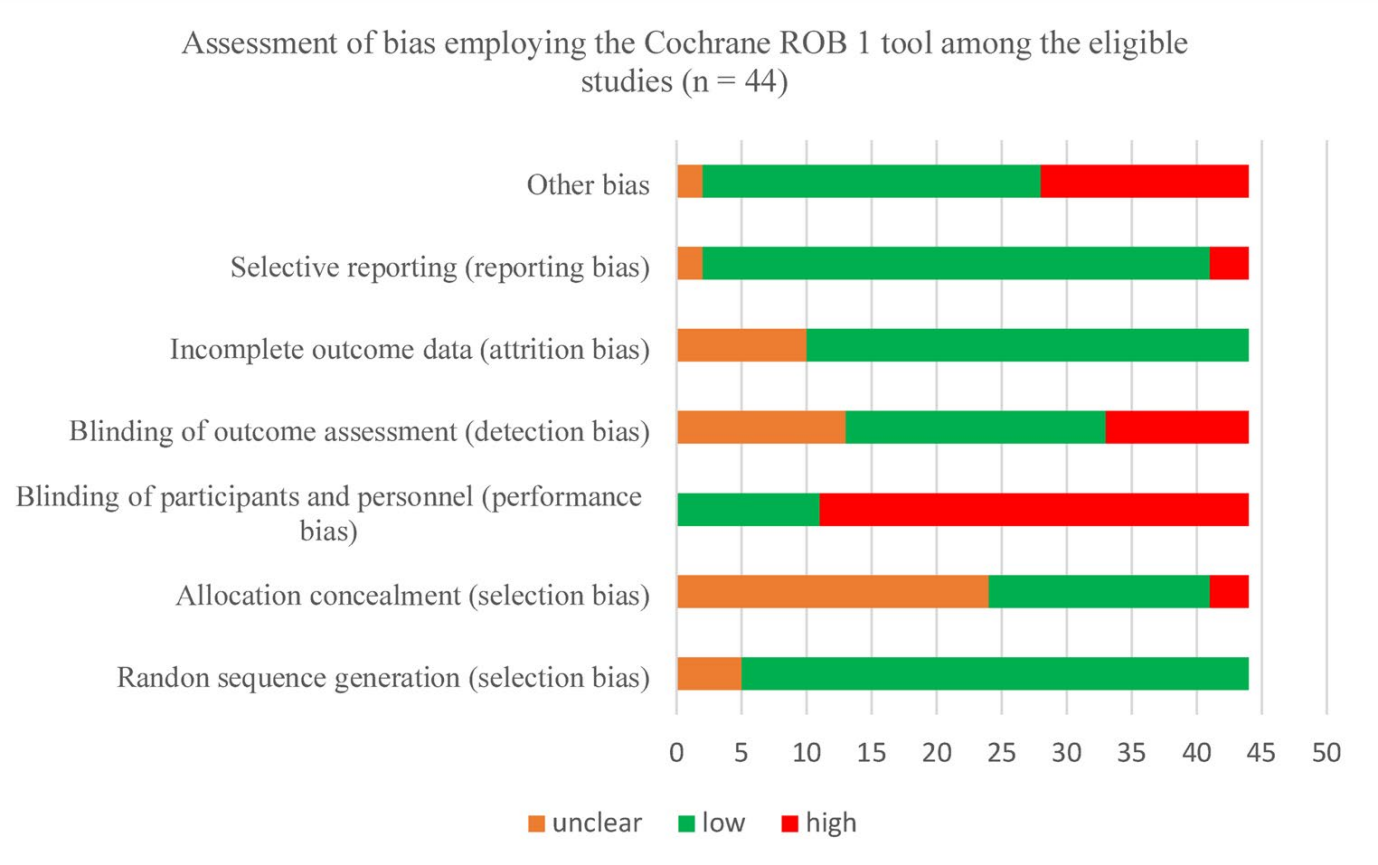
Cochrane Collaboration Risk of Bias 1 Tool Assessment of Identified RCTs (*n* = 44)

Screening was managed using Covidence^®^ and EndNote^®^ 20 software. In total, 5,529 studies and 42 studies were identified via the RLR and Catenacci et al., respectively (*n* = 5,571). A total of 1,577 duplicates were removed. Title and abstract screening resulted in the elimination of 3,752 studies. Thus, 242 studies were eligible for full-text assessment. The full-text assessment identified a total of 44 RCTs eligible for inclusion in the data-extraction and narrative review. The PRISMA flow, which details the reasons for inclusion and exclusion, is illustrated in Appendix (Fig. 1). The included RCTs are summarized in Table 1.

### Data Extraction

The 44 identified RCTs, underwent data extraction in line with the prespecified data collection template. The data extracted is presented in Table 1. The 44 RCTs are from a broad range of jurisdictions; from Asia [40–47] (specifically Japan [48–57], Korea [58–63], China), from Europe (specifically France and Germany [64–67]), UK [68], in addition to multinational trials across multiple continents [64–67, 69–79]. Table 1 illustrates that, for each RCT, the study title, author and year of publication along with the number of participants in each arm (range from 15 to 357 participants) was recorded. Across the 44 RCTs, 37 investigated treatments in the second- line setting, five were in the second- and later-line setting and two were in the third- and later-line setting. As advised by clinical expert opinion, prior first-line treatments, across the eligible RCTs, were categorized as: fluoropyrimidine alone; fluoropyrimidine + platinum compounds; fluoropyrimidine + platinum compounds + trastuzumab (in the HER2-positive setting); fluoropyrimidine + platinum compounds or taxane-based chemotherapy; fluoropyrimidine + platinum compounds or anthracycline or docetaxel. These prior treatment categories are described in the data extraction table.

### Narrative Review of Eligible Randomised Controlled Trials (*n* = 44)

#### Second-line Setting (37 RCTs)

The 37 eligible RCTS, in this setting, were a mixture of Phase II RCTs (*n* = 16), Phase III RCTs (*n* = 18), RCTs encompassing both Phases II and III (*n* = 2) and one trial that did not report the phase. Of the 37 RCTS, eight implemented a double-blind design [46, 47, 58, 70, 72–75]; the remaining were open-label trials. Of the 37 RCTs, 35 compared the intervention of interest to an active comparator; two were placebo-controlled [68, 72]. Populations across the trials were, in the main, heterogeneous. Certain studies were country specific, a number compared Asian and western populations, and several studies included a global population with Asian subgroups.

##### Paclitaxel Versus Paclitaxel in Combination with Various Treatments

A number of RCTs assessed the efficacy of paclitaxel alone versus paclitaxel in combination with various treatments. No statistically significant differences were observed for napabucasin + paclitaxel versus paclitaxel for OS, PFS and ORR [70]. Likewise, raltitrexed + paclitaxel was not associated with statistically significant improvements in OS, PFS and ORR versus paclitaxel [80]. Similarly, trastuzumab + paclitaxel did not improve OS, PFS and ORR versus paclitaxel alone [48]. Adding olaparib to paclitaxel did not lead to a statistically significant improvement in PFS, but did lead to a statistically significant OS benefit [58]. Olaparib + paclitaxel was not associated with a statistically significant OS benefit versus paclitaxel alone [47]. In the RAINBOW trial, OS was statistically significantly longer with ramucirumab + paclitaxel than with paclitaxel alone [73]. Muro et al. reported on a subgroup analysis of the RAINBOW trial which comprised participants from East Asia. Ramucirumab + paclitaxel was associated with a statistically significant PFS benefit, but not with a statistically significant OS benefit, versus paclitaxel alone [46]. Also, Shitara et al. concluded that OS and PFS outcomes associated with ramucirumab + paclitaxel were similar between Japanese and Western subgroups of RAINBOW [74]. Satoh et al. concluded that lapatinib + paclitaxel did not statistically significantly improve OS or PFS, versus paclitaxel, in an Asian population; however, a significant ORR benefit was seen [44]. Paclitaxel + S-1 was not associated with a statistically significant benefit in OS, PFS or ORR, versus paclitaxel, in an Asian population [53].

##### Paclitaxel Versus Other Treatments

Paclitaxel has also been compared to other treatments. Paclitaxel was associated with a significant PFS benefit, but not with a significant OS benefit, versus 5-FU [54]. AZD4547 (a selective FGFR-1, 2, 3 tyrosine kinase inhibitor) did not significantly improve PFS, relative to paclitaxel alone, in patients with gastric cancer displaying FGFR2 polysomy or gene amplification [76]. Two studies evaluated paclitaxel versus irinotecan; in both no statistically significant differences were observed between arms for PFS and OS [49, 61]. In their four-arm study, Kawase et al., compared irinotecan-alone, paclitaxel-alone, irinotecan + S-1 and paclitaxel + S-1. A significant benefit was seen for PFS, but not for OS, when comparing paclitaxel-alone versus irinotecan alone. No PFS benefit or OS benefit was seen in the S-1 combination groups versus the monotherapy groups [41]. No PFS benefit or OS benefit was seen in the S-1 combination groups versus the monotherapy groups [41]. In three RCTs, pembrolizumab was evaluated versus paclitaxel; pembrolizumab was not associated with a statistically significant OS benefit in any of these RCTs [40, 69, 77].

Kang et al. concluded that DHP107 (a novel oral formulation of paclitaxel) was noninferior to paclitaxel for OS, PFS and ORR [62]. Likewise, Shitara et al. concluded that nab-paclitaxel (nanoparticle albumin-bound paclitaxel) was non-inferior to paclitaxel, for OS [55].

##### Docetaxel Versus Other Treatments

In the COUGAR-02 trial, the addition of docetaxel to active-symptom-control was associated with a statistically significant improvement in OS [68]. Yi et al. concluded that the adding sunitinib to docetaxel did not result in a statistically significant improvement in the time to progression [42]. However, elsewhere, the addition of oxaliplatin to docetaxel was associated with a statistically significant improvement in PFS [59]. Lee et al. concluded that the addition of S-1 to docetaxel resulted in a statistically significant PFS benefit but that the addition of cisplatin to docetaxel did not improve PFS [60].

In the GATSBY trial, trastuzumab emtansine was not associated with a statistically significant OS benefit when compared to physician-choice taxane (either paclitaxel or docetaxel) in individuals with HER-2 positive advanced gastric [75].

Two studies compared cisplatin + irinotecan to irinotecan; with no OS benefit was demonstrated in either [50, 52]. A statistically significantly prolonged PFS was demonstrated in one of these RCTs [50], but not in the other [52].

Five RCTs evaluated irinotecan versus different comparators. In an RCT that compared irinotecan alone to BSC, irinotecan alone was associated with a statistically significant OS benefit [65]. There were no statistical significant differences observed in OS, PFS and ORR between mFOLFIRI (irinotecan + 5-FU + leucovorin) and irinotecan alone [43]. In their three-arm trial, Roy et al. compared PEP02 (a liposomal nanocarrier formulation of irinotecan) to irinotecan alone and to docetaxel alone. No statistically significant differences in OS, PFS and ORR were noted between the three arms [71]. In another RCT there was no statistically significant difference observed in OS between S-1 + irinotecan and irinotecan alone [51]. The addition of nimotuzumab to irinotecan was not associated with a statistically significant improvement in OS and PFS versus irinotecan alone [45].

##### **Targeted Treatments & Immunotherapy Treatments**

Fuchs et al. concluded that ramucirumab + BSC was associated with a statistically significant OS benefit versus placebo + BSC [72]. Tougeron et al. randomised participants to receive FOLFIRI + durvalumab or FOLFIRI + durvalumab + tremelimumab. No statistically significant differences in OS, PFS or ORR were observed between the two arms [81].

Lorenzen et al. concluded that treatment with FOLFIRI + ramucirumab resulted in similar OS, but improved ORRs and PFS outcomes relative to those of paclitaxel + ramucirumab; however, no p-values were reported [64].

Yan et al. concluded that intermittent-apatinib + docetaxel was associated with similar OS, PFS and ORR outcomes to those of continuous-apatinib + docetaxel, with no statistically significant differences identified between the two treatment regimens [82]. In another RCT, it was concluded that toripalimab + apatinib did not improve OS, PFS and ORR compared with physician’s-choice chemotherapy (defined as either irinotecan or paclitaxel or docetaxel) [83].

Of the 37 RCTs in this setting, 19 reported event rates for Grade ≥ 3 TRAEs for the intervention versus the comparator. None of these 19 RCTs reported any statistically significant differences in Grade ≥ 3 TRAEs in the intervention arms relative to the comparator.

#### Second- and Later-Line Setting (*n* = 5 RCTs)

In this setting, three RCTs were Phase II [56, 57, 67] and two were Phase III [63, 66]. Two implemented a double-blind design [66, 67]; three were open-label [56, 57, 63]. Populations across the trials were considered to be heterogeneous. Various studies were conducted in Germany, Korea and Japan.

In their RCT, Lorenzen et al. concluded that paclitaxel + everolimus did not improve OS, PFS and ORR outcomes compared with paclitaxel [66]. Fushida et al. reported no statistically significant differences between paclitaxel + valproic acid and paclitaxel for both OS and PFS [57]. Shitara et al. reported that dose-escalated paclitaxel did not result in a statistically significant improvement in OS or ORR compared to standard dose of paclitaxel. However, PFS was statistically significantly prolonged with dose-escalated paclitaxel [56]. Kang et al. concluded that chemotherapy (either irinotecan or docetaxel) + BSC statistically significantly improved OS compared to BSC alone [63]. Moehler et al. found that the addition of sunitinib (an anti-angiogenic receptor tyrosine kinase inhibitor) to FOLFIRI did not result in statistical significant improvements in PFS, OS, or ORR outcomes compared to FOLFIRI alone [67].

Only one of the RCTs in this setting reported event rates for Grade ≥ 3 TRAEs; no statistically significant difference between arms was detected [66].

#### Third- and Later-line Setting (*n* = 2 RCTs)

Shitara et al., 2018 evaluated the efficacy of trifluridine-tipiracil compared to BSC in individuals who had previously received two or more standard-of care-regimens. Trifluridine-tipiracil was associated with a statistically significant benefit in OS, and PFS but not in ORR. Grade ≥ 3 TRAEs were reported but statistical analyses were not presented.

Shitara et al., 2020 evaluated trastuzumab deruxtecan [79] as compared with physician-choice chemotherapy (irinotecan (89%) and paclitaxel (11%)) in individuals with HER-2 positive gastric cancer who had received a median of two previous systemic treatments. Trastuzumab deruxtecan was associated with a statistically significant benefit in both OS and PFS. No statistically significant differences, between arms, were detected in ORR and Grade ≥ 3 TRAEs.

### Assessment of Risk of Bias

All 44 identified RCTS were subjected to a RoB assessment using the Cochrane Collaboration RoB 1 Tool for RCTs. Most RCTs were open-label in design, utilized an intention-to-treat analysis approach, and all were generally well-balanced across treatment arms for baseline characteristics.

The Y-axis describes the seven domains under consideration. The X-axis describes the number of RCTs that comply (green) or do not comply (red) with each domain or where reporting is unclear (amber).

The RoB 1 Tool assessment outcomes are described in Fig. 1. The Y-axis describes the seven domains considered within the Tool. The X-axis describes the number of studies that comply (green) or do not comply (red) with each domain, or where reporting is unclear (amber). As illustrated, no RCTs were compliant with all seven domains. Amongst all RCTs, compliance with certain domains was high. For example, methods used to generate the allocation sequence and reporting bias were adequately described in 39 RCTs. Attrition bias was adequately described in 34. Amongst all RCTs, compliance with certain domains was low. For example, across 33 RCTs, performance bias or measures used to blind trial participants and researchers from knowledge of which intervention a participant received were, in the main, poorly described. In 24, the allocation-concealment approaches used were not described in sufficient detail. A detailed, domain-level risk of bias table for all individual RCTs (*n* = 44) is presented in Appendix Table 2.

### Data Synthesis and Analysis

#### Construction of Evidence Networks

All 44 identified RCTs, across all treatment-line settings, were assessed for potential inclusion in the four outcome-specific evidence networks. The assessment-process flow and conclusions, for each outcome-specific network, are described in Appendix (Figs. 6, 7, 8 and 9).

Briefly, only those RCTs that contributed to a path connecting two or more treatments of interest were included. RCTs were necessarily excluded from the OS and PFS networks when HRs were not reported. Likewise, RCTs were excluded from the ORR and Grade ≥ 3 TRAEs networks when event rates or absolute number of events were not reported.

The exchangeability of remaining RCTs was then assessed. Any RCT that differed substantially from the main study pool in terms of participant inclusion and exclusion criteria, biomarker status and prior treatments were excluded from the final evidence networks.

This assessment process concluded that it was feasible to construct evidence networks for each outcome of interest (OS, PFS, ORR and Grade ≥ 3 TRAEs) in the second-line setting. Also, that it was not feasible to construct evidence networks, for any of the outcomes, in the second- and later-line setting or in the third- and later-line setting.

### Network Meta-Analysis in the Second-Line Setting

#### Overall Survival

**Fig. 2:**
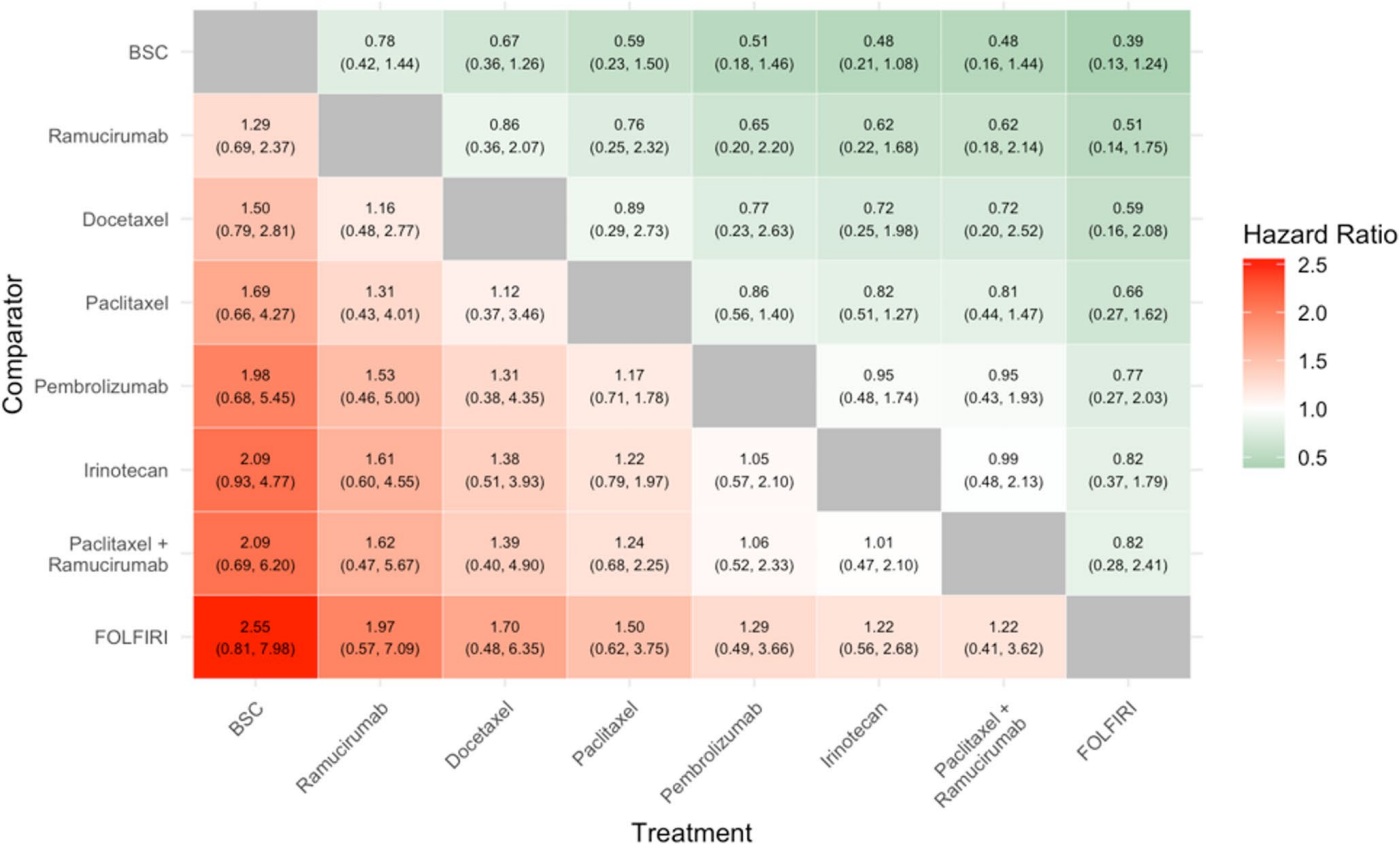
Results of NMA for Overall Survival

Nine RCTs, comprising eight treatments of interest, were eligible for inclusion in the evidence network for OS. The treatments were paclitaxel alone, docetaxel alone, irinotecan alone, FOLFIRI, ramucirumab, ramucirumab + paclitaxel, pembrolizumab and BSC (see Appendix (Fig. 2)).

The relative efficacy estimates (and associated uncertainty) obtained from the NMA for all pairs of included treatments are presented in Fig. 2. As shown, there are no statistically significant differences in the risk of an OS event (death) across any of the pair-wise comparisons. However, certain trends are observed. For example, BSC exhibits a numerically (but not statistically significant) higher risk of death relative to all other comparators. Likewise, FOLFIRI exhibits a numerically lower risk of death relative to all other comparators included in this evidence network.

The HR (95%CrI) for each pair-wise comparison for the treatment (X-axis) vs. the comparator (Y-axis) is described, with values of less than one indicating a lower risk of death with the treatment relative to the comparator. For example, the HR (paclitaxel vs. pembrolizumab) = 1.17 (95%CrI 0.71, 1.78), indicating a numerically (but not statistically significant) higher risk of death with paclitaxel.

Green indicates a point-estimate that favours the treatment; red indicates a point estimate that favours the comparator. The colour intensity indicates the magnitude of the point estimate (but does not indicate statistical significance or width of the 95%CrI).

Where BSC = Best supportive care, HR = Hazard ratio, CrI = Credible Interval, FOLFIRI = Folinic acid Fluorouracil Irinotecan.

#### Progression-Free Survival

**Fig. 3:**
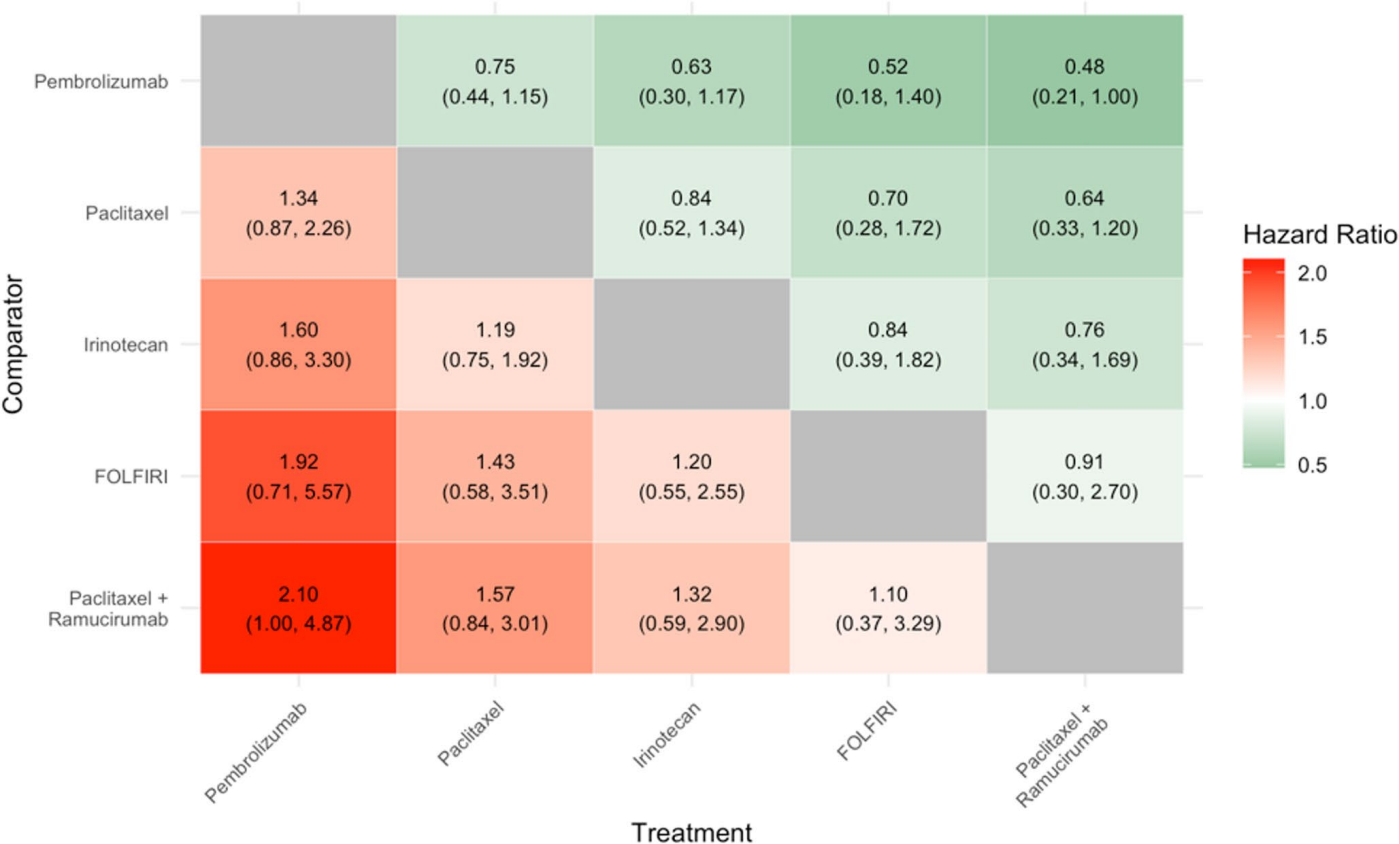
Results of NMA for Progression Free Survival

Six RCTs, comprising five treatments, were eligible for inclusion, in the evidence network for PFS. The treatments were: paclitaxel alone, irinotecan alone, FOLFIRI, ramucirumab + paclitaxel and pembrolizumab (see Appendix (Fig. 3)).

The results of the NMA for PFS are shown in Fig. 3. No statistically significant differences in efficacy between treatments were observed for this outcome. Also, it can be seen, for example, that Pembrolizumab is associated with a numerically (but not statistically significant) higher risk of progression or death versus each of the treatments in the network. Likewise, ramucirumab + paclitaxel is associated with a numerically lower risk of progression or death versus each treatment in the network.

The HR (95%CrI) for each pair-wise comparison for the treatment (X-axis) vs. the comparator (Y-axis) is described, with values of less than one indicating a lower risk of death with the treatment relative to the comparator. For example, the HR (irinotecan vs. FOLFIRI) = 1.20 (95%Ci 0.55, 2.55), indicating a numerically (but not statistically significant) higher risk of PFS with irinotecan.

Green indicates a point-estimate that favours the treatment; red indicates a point estimate that favours the comparator. The colour intensity indicates the magnitude of the point estimate (but does not indicate statistical significance or width of the 95%CrI).

The upper 95%CrI (ramucirumab + paclitaxel vs. pembrolizumab) = 1.0007; the lower 95%CrI (pembrolizumab versus ramucirumab + paclitaxel) = 0.9993. Both values are rounded to 1.0 in the table.

#### Objective Response Rate

**Fig. 4:**
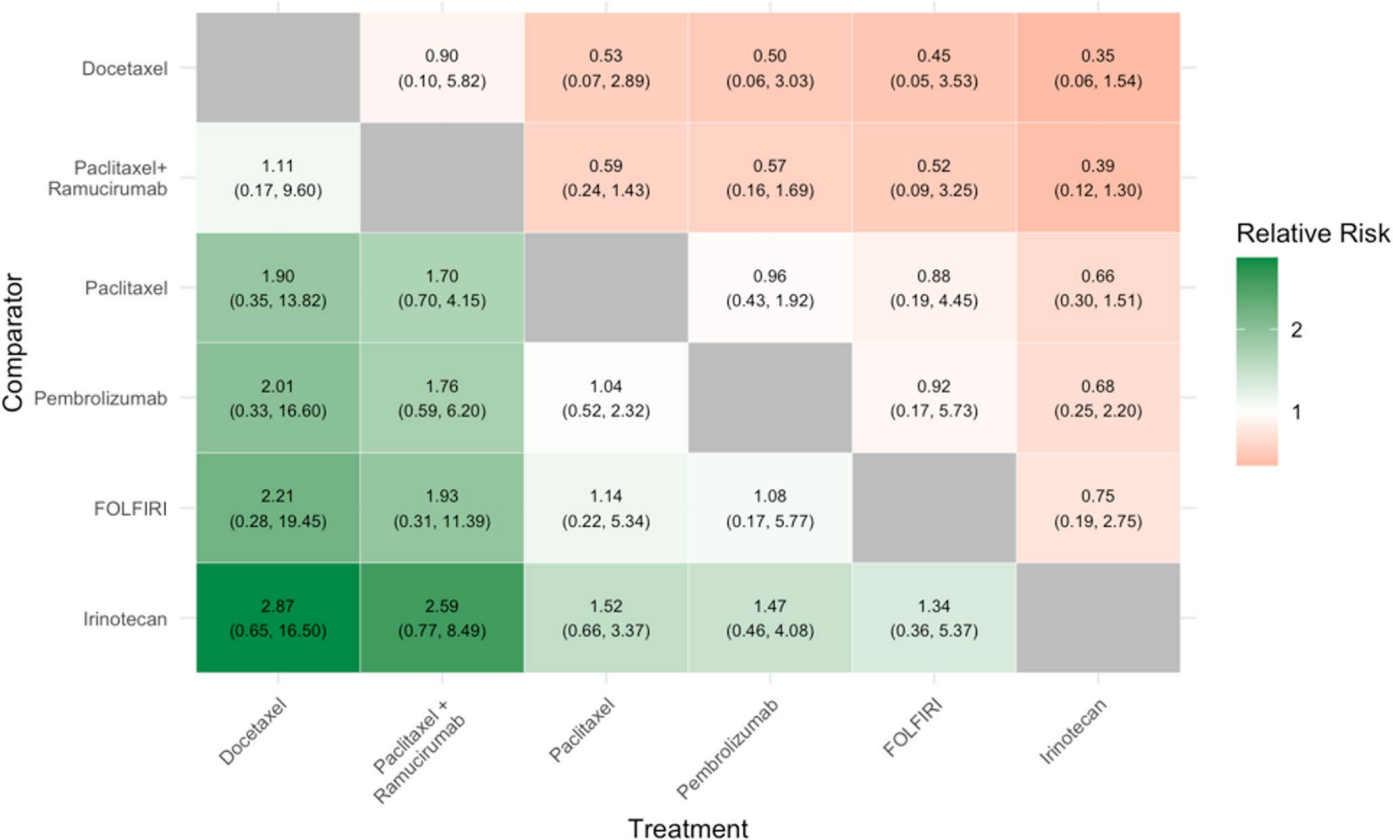
Results of NMA for Objective Response Rate

Eight RCTs, pertaining to six treatments, (see Appendix (Fig. 4)) were eligible for inclusion in the ORR evidence network. The results of the NMA for this outcome (Fig. 4) show that, across all pair-wise comparisons, there are no instances of statistically significant differences in ORR. Estimated ORR is numerically higher with docetaxel versus all other treatments in the network, and lower with irinotecan.

The RR (95%CrI) for each pair-wise comparison for the treatment (X-axis) vs. the comparator (Y-axis) is described, with values greater than 1 indicating a higher rate of objective response with the treatment relative to the comparator. For example, the RR (docetaxel vs. FOLFIRI) = 2.21 (95%CrI 0.28, 19.45), indicating a numerically (but not statistically significant) higher rate of objective response (tumour shrinkage) with docetaxel compared to FOLFIRI.

Green indicates a point-estimate that favours the treatment; red indicates a point estimate that favours the comparator. The colour intensity indicates the magnitude of the point estimate (but does not indicate statistical significance or width of the 95%CrI).

#### Grade ≥ 3 Treatment Related Adverse Events

**Fig. 5:**
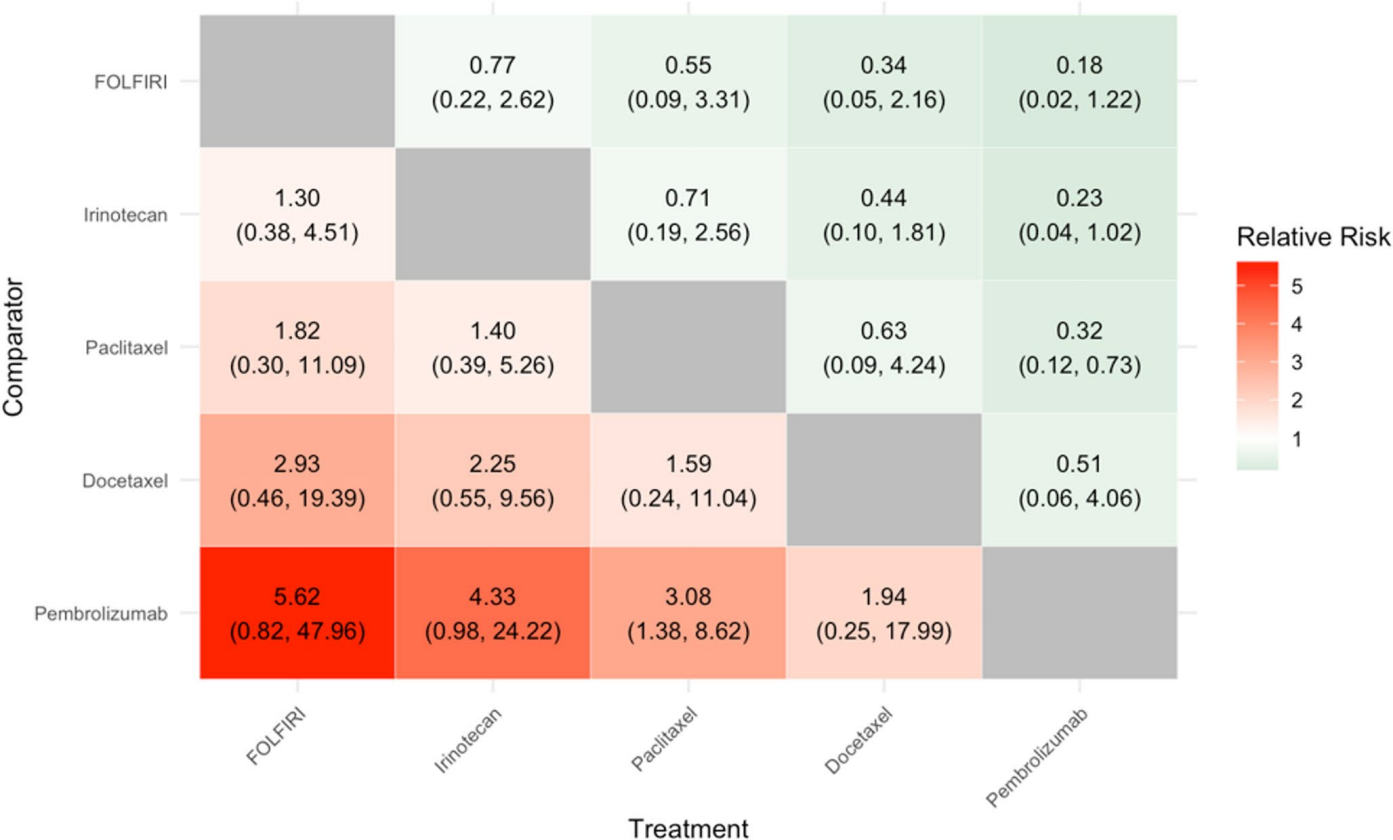
Results of NMA for Grade ≥ 3 TRAEs

A total of five studies, pertaining to five treatments, were eligible for inclusion in the Grade ≥ 3 TRAEs evidence network (see Appendix (Fig. 5)).

The results of the NMA show that the only statistically significant finding is the decreased risk of Grade ≥ 3 TRAEs for pembrolizumab versus paclitaxel. It highlights also that FOLFIRI is associated with a numerically higher (but not statistically significant) risk of Grade ≥ 3 TRAEs versus all other treatments in the network. Likewise, pembrolizumab is associated with a lower risk of Grade ≥ 3 TRAEs versus all comparators; significance was only identified in the comparison versus paclitaxel. In general, confidence intervals for this outcome were wide due to low event numbers.

The RR (95%CrI) for each pair-wise comparison for the treatment (X-axis) vs. the comparator (Y-axis) is described, with values greater than 1 indicating a higher rate of adverse events with the treatment relative to the comparator. For example, the RR (paclitaxel vs. docetaxel) = 1.59 (95%CrI 0.24, 11.04), indicating a numerically (but not statistically significant) higher rate of adverse events with paclitaxel compared to docetaxel.

Green indicates a point-estimate that favours the treatment; red indicates a point estimate that favours the comparator. The colour intensity indicates the magnitude of the point estimate (but does not indicate statistical significance or width of the 95%CrI).

## Discussion

This work involved the identification of relevant RCTs that investigated the clinical efficacy and safety of eleven treatments for advanced and metastatic gastric cancer, in the second- and later-line setting, as recommended by both ESMO and NCCN guidelines. The subsequent implementation of evidence synthesis networks allowed investigation of the relative efficacy and safety of these treatments. Primary outcomes of interest were OS and PFS; additional outcomes of interest were ORR and Grade ≥ 3 TRAEs.

In total 44 RCTs were deemed to be eligible for inclusion in the data-extraction and narrative review. This review provided useful insights into the research undertaken, to date, in this setting. It is noted, for example, that the 44 RCTs are from a broad range of jurisdictions. The majority of RCTs (*n* = 37) investigated treatments in the second-line setting. Between-trial heterogeneity in several key criteria (including number of participants, trial phase, previous treatments, blinding methods and patient demographics) is evident. The RCTs were associated with varying risks of bias. Differences were noted, across the RCTs, in the outcomes investigated. Not all RCTs, for example, considered the efficacy (OS, PFS and ORR) and safety (Grade ≥ 3 TRAEs) outcomes of interest here.

An understanding of the relative efficacy and safety, of all the treatments of interest, required the implementation of NMAs. Given the data availability, NMAs were feasible in the second-line setting only and only for certain treatments. Eight treatments could be included in the OS NMA, five treatments in the PFS NMA and six in the ORR NMA. No statistically significant differences in the efficacy of the treatments across each of these networks were identified; however, certain trends were noted. For example, the estimated HRs suggest that mortality may be higher with BSC compared to all other treatments in the OS network, and lower with FOLFIRI. In terms of PFS, the risk of progression or death may be higher with pembrolizumab relative to the other comparators, and lower with ramucirumab + paclitaxel. Estimated rates of objective response were highest with docetaxel and lowest with irinotecan. Five treatments could be included in the Grade ≥ 3 TRAEs NMA; this indicated that pembrolizumab is associated with a statistically significant decreased risk of Grade ≥ 3 TRAEs versus paclitaxel. No other statistically significant differences identified in the risk of Grade ≥ 3 TRAEs across the treatments in this network, and confidence intervals were wide due to low event numbers overall.

This work has investigated the relative efficacy (in terms of a number of key outcomes) along with safety of a wide range of treatments for advanced and metastatic gastric cancer, in the second-line setting. To our knowledge, this is the first NMA which has specifically considered treatments as recommended by both ESMO and NCCN. This NMA adds to previously published work, in the second-line setting. In their NMA, Badiani et al. [84] included eligible literature, up to February 2015, for six treatments. OS was the outcome of interest. Badiani et al. concluded, from their NMA, that ramucirumab + paclitaxel is associated with a statistically significant decreased OS-event risk versus each of irinotecan, paclitaxel, docetaxel, ramucirumab, and BSC [84]. Although our OS NMA outputs are directionally consistent with that of Badiani et al., none of our outputs were statistically significant. The differences in findings can, at least partly, be explained by key methodological and evidence-base differences in the approaches taken by Badiani et al. and our group. Badiani et al. used median OS from seven RCTs (published to Feb 2015) [84]. Median survival does not account for censoring or the shape of the time-to-event curve and can therefore distort relative effects in indirect comparisons (Guyot et al., 2012; Michiels et al., 2005). Our NMA instead used the HR which is the recommended measure for time-to-event outcomes. Further, we incorporated RCTs published to May 2024.

In their 2019 NMA, Cheng et al. ranked treatments for efficacy and safety effects. Amongst the findings, it was concluded that paclitaxel + olaparib and ramucirumab + paclitaxel dominated the OS ranking, and ramucirumab + paclitaxel the PFS ranking, however, these rankings and the underlying effect estimates were uncertain [85].

An evidence network was not feasible, in the present study, for the third- and later-line setting, Zhang et al. 2023 [86], however, could construct an evidence network for third-line treatments given that their PICOS differed to ours. For example, their PICO included treatments that are not recommended by ESMO and NCCN. Their work indicated that trastuzumab deruxtecan was associated with a statistically significant improvement in OS compared to chemotherapy [86].

The methodology employed for this research is robust, being aligned with best-practice guidance [33, 34]. The outputs of this work are of interest to all healthcare decision makers including policy makers, health payers, clinicians, patients, carers and families.

The limitations of this work should be noted. Given data availability, it was not possible to investigate the relative efficacy and safety of treatments for use in the third- and later-line setting. Also, it was not possible to include all treatments of interest within each of the efficacy and safety NMAs in the second-line setting. The inclusion of pembrolizumab in our networks is noted. ESMO and NCCN Guidelines recommend pembrolizumab in the setting of MSI-H/dMMR tumours [7, 22]. NCCN also recommends use in the setting of TMB-high tumours [22]. The RCTs in the second-line setting (KEYNOTE-061 and KEYNOTE-063), which informed the pembrolizumab node in our evidence networks, mainly recruited patients with PD-L1-positive disease and this was regardless of MSI-H/dMMR and TMB status. In KEYNOTE-061, a very small subgroup (5.3%) had positive MSI-H status [8]. MSI-H status was not a trial stratification factor, and thus analyses in this subgroup are at a risk of bias [77]. Also, a comparison of outcomes for this KEYNOTE-061 subgroup with those of the full populations from the other RCTs, would require the strong assumption that MSI-H status is an effect modifier for pembrolizumab versus paclitaxel, but not for other comparisons in the network. We note the MSI-H/dMMR and TMB-high status of patients in KEYNOTE-063 is not provided [40]. Thus, it is not possible to consider pembrolizumab in the settings of MSI-H/dMMR tumours and TMB-high tumours only within our NMAs. Further work is warranted here.

Further, an important consideration in interpreting the results of this NMA is the fact that many comparisons yielded wide credible intervals for relative treatment effects and were not statistically significant. This can be seen, for example, in Fig. 2 which describes the outputs of the NMA for OS for each of the comparisons versus BSC. Here all RR point estimates, for each intervention versus BSC, show that each intervention is associated with a numerical benefit in OS versus BSC. However, in each instance the credible intervals are particularly wide. This is likely to be partially a consequence of the low event rates and small sample sizes across many included RCTs, as well as the between-study heterogeneity and reliance on indirect evidence for some comparisons (which can lead to lower statistical precision). Therefore, it is possible that true differences in efficacy and/or safety between certain treatments exist but were not detected due to low statistical power (i.e., a type II error). Despite the lack of statistical significance, the results of the NMA indicate trends in the data, and further research is suggested to identify optimal treatments.

We acknowledge that the generalizability of our results may be somewhat limited given that several trials were conducted entirely in East Asian populations or included subpopulations from East Asia. Our group considered that undertaking scenario analysis to exclude such studies from the networks would have been of interest. However, unfortunately, it was not possible to undertake robust scenario analysis here, given the relatively small number of trials in our networks. Also, we have restricted our SLR to English-language full texts. This may have introduced selection bias and an under-representation of certain regions or regimens.

Future NMAs should incorporate additional on-going and planned RCTs to enhance the precision of effect estimates and provide more definitive evidence regarding the comparative efficacy and safety of all treatments of interest here. Of note, the treatments of interest, in this work, are defined as those recommended by both the ESMO and NCCN Guidelines. As such, it is beyond the scope, here, to include novel treatments, in this setting, of which there are a number. It is planned that, in time, our evidence networks, will be updated to include emerging data from RCTs of novel treatments. This will allow investigations of relative efficacy and safety of these novel treatments versus the treatments in our networks. In line with robust practice, this will be feasible, only, if such novel treatments are supported by RCTs that are interchangeable with those in our networks. Also, the certainty of the resultant NMA outputs will be improved where the RCT sample sizes are sufficient [33].

## Conclusion

The appreciable number of RCTs captured in our search highlights that the treatment landscape for advanced and metastatic gastric cancer, in the second- and later-line setting, continues to evolve. Implementation of NMAs were feasible in the second-line setting only and only for certain treatments. NMA outputs suggest that the introduction of novel treatments, in this setting, have not had a statistically significant impact on key efficacy outcomes, and have had little impact on safety outcomes, versus more established treatments. There remains a need for novel treatments that will have a significant beneficial impact on both efficacy and safety outcomes in this setting.

## Supplementary Information

Below is the link to the electronic supplementary material.

Supplementary Material 1 (DOCX 376 KB)

## Data Availability

Data is provided within the manuscript.
