# Supplementary material for: Relative Clinical Efficacy and Safety of Second- or Later-Line Treatments for Advanced and Metastatic Gastric Cancer: A Rapid Review and Network Meta-Analysis
Source: J Gastrointest Cancer. 2026 Feb 6;57(1):36. doi: 10.1007/s12029-026-01407-z (PMC12881065; doi:10.1007/s12029-026-01407-z)
Supplement: Supplementary file 1 — Supplementary Material 1 (DOCX 376 KB) [file 12029_2026_1407_MOESM1_ESM.docx]

**Table 1: PICOS framework**

| Review Question: What are the published randomised controlled trials of systemic treatments for advanced and metastatic gastric cancer in the second- or later-line setting? | |
| --- | --- |
| Population | Individuals with a diagnosis of advanced or metastatic gastric cancer in the second- or later-line setting. |
| Intervention | All systemic treatments approved by both ESMO (Lordick et al., 2022) and NCCN Guidelines (Ajani et al., 2022) and that are also aligned with Expert Clinical opinion.  Studies evaluating at least one of the following treatments:   - Paclitaxel - Docetaxel - Irinotecan - FOLFIRI - Ramucirumab - Ramucirumab + Paclitaxel - Pembrolizumab - Trastuzumab deruxtecan (for HER2 overexpression-positive adenocarcinoma) - Dostarlimab - Entrectinib - trifluridine-tipiracil - Best supportive care |
| Comparator | Unrestricted |
| Outcomes | Primary Outcomes: Overall survival, progression-free survival. Additional outcomes: overall response rate, Grade ≥ 3 Treatment-related adverse events |
| Study design | randomised controlled trials |
| Exclusion criteria | Studies in first-line treatment for advanced and metastatic gastric cancer, conference papers, commentaries, abstracts, and letters, where the full paper was unobtainable.  Subgroup, exploratory and post-hoc analysis of original trials. |
| Language | English only |
| Time period | 2009 to 29 May 2024 |

*ESMO: European Society for Medical Oncology; NCCN: National Comprehensive Cancer Network

**Fig 1: PRISMA flow chart**

**
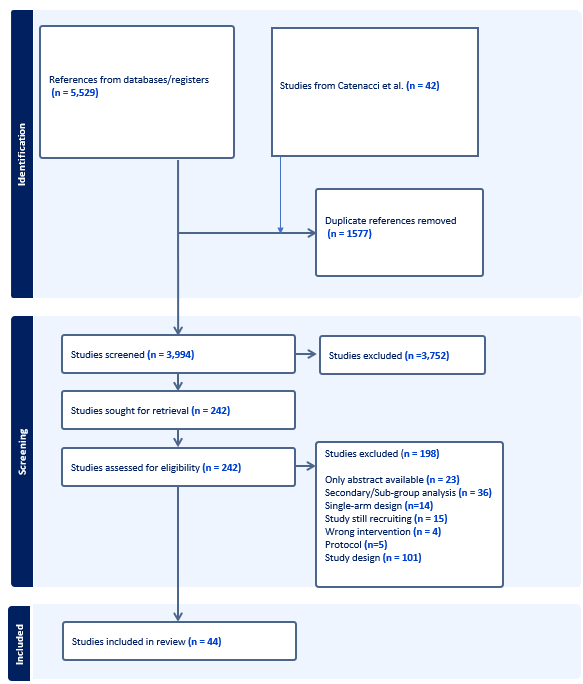
**

Fig 2: Bayesian Network Meta-Analysis of Overall Survival


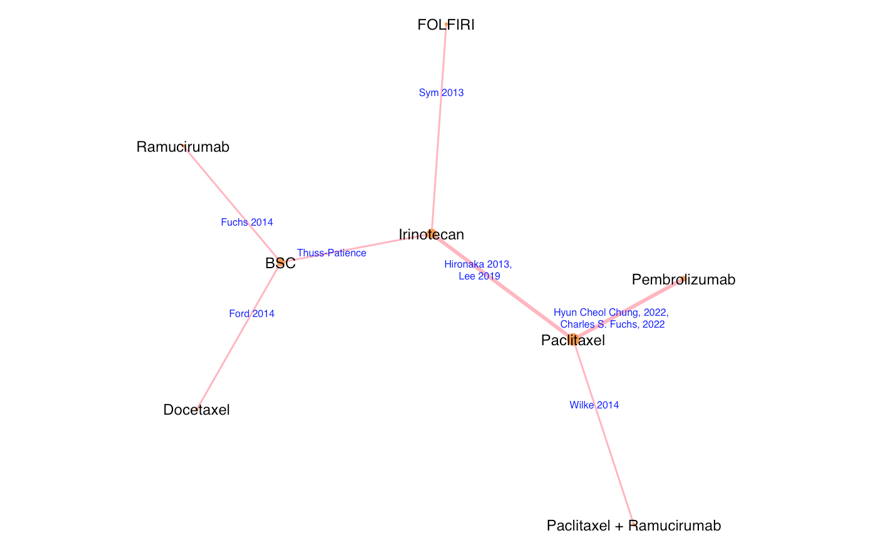

Fig 3: Bayesian Network Meta-Analysis of Progression Free Survival


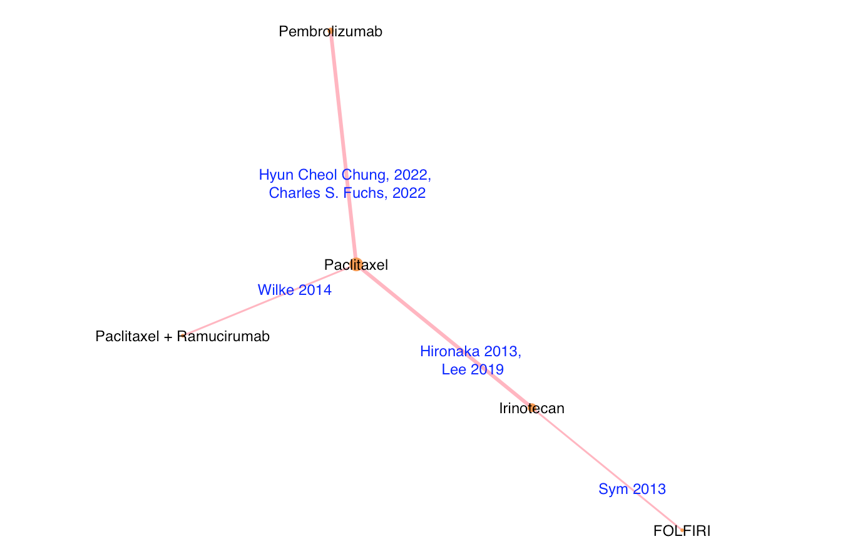


Fig 4: Bayesian Network Meta-Analysis of Objective Response Rate


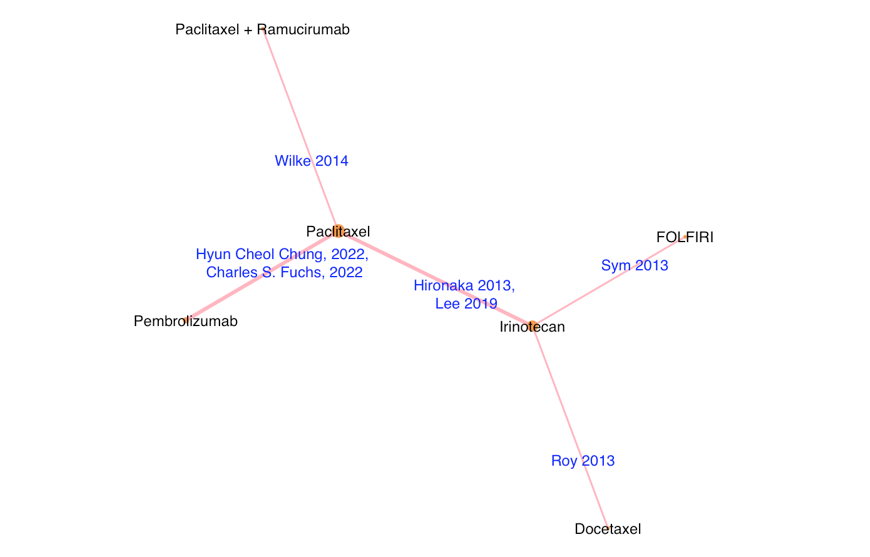


Fig 5: Bayesian Network Meta-Analysis of Grade ≥ 3 TRAEs


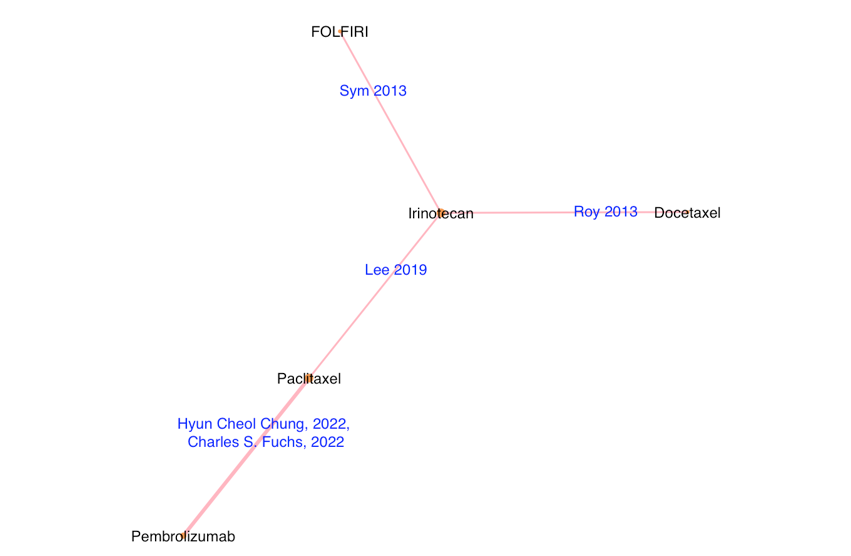


**Fig 6: Flow chart depicting the process of exclusion and final inclusion of studies in the OS network**


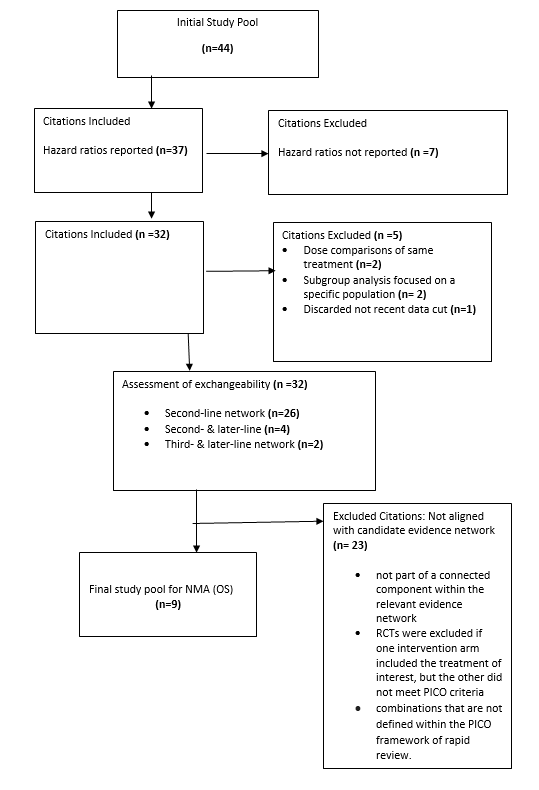


**Fig 7: Flow chart depicting the process of exclusion and final inclusion of studies in the PFS network**


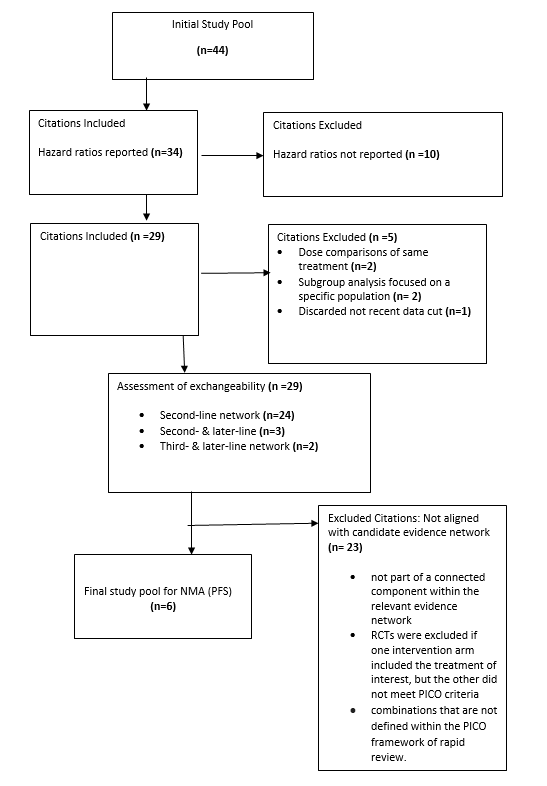


**Fig 8: Flow chart depicting the process of exclusion and final inclusion of studies in the ORR network**


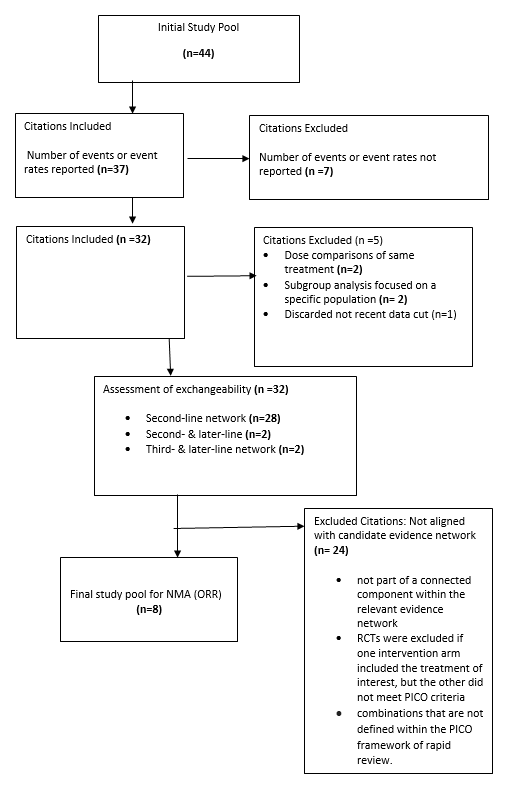


**Fig 9: Flow chart depicting the process of exclusion and final inclusion of studies in the Grade ≥ TRAEs network**

**
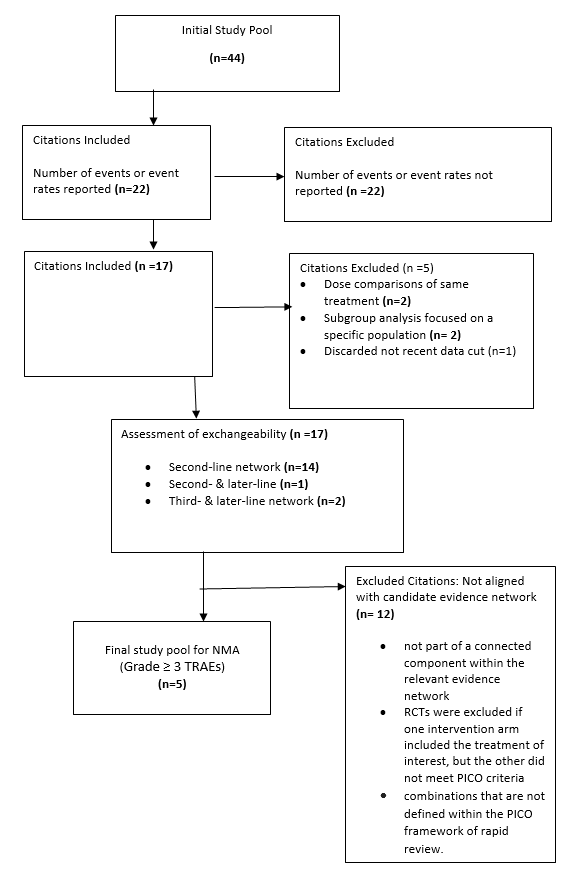
**

**Table 2: Domain-Level Risk of Bias Assessment for Individual Identified RCTs (n=44) using Cochrane Collaboration Risk of Bias 1 Tool**

| Study | Randon sequence generation (selection bias) | Allocation concealment (selection bias) | Blinding of participants and personnel (performance bias) | Blinding of outcome assessment (detection bias) | Incomplete outcome data (attrition bias) | Selective reporting (reporting bias) | Other bias |
| --- | --- | --- | --- | --- | --- | --- | --- |
| **Second-line Setting (n=37)** | | | | | | | |
| Paclitaxel versus Paclitaxel in Combination with Various Treatments | | | | | | | |
| Shah et al (2022) | L | L | L | L | U | L | L |
| Zhao et al (2023) | U | H | H | L | U | L | L |
| Makiyama et al (2020) | L | U | H | L | L | L | L |
| Bang et al (2015) | L | L | L | L | L | L | L |
| Bang et al (2017) | L | L | L | L | L | L | L |
| Wilke et al (2014) | L | L | L | L | L | L | H |
| Muro et al (2016) | L | L | L | L | L | H | H |
| Shitara et al (2016) | L | L | L | L | L | H | H |
| Satoh et al (2014) | L | L | H | U | L | L | L |
| Nakanishi et al (2016) | L | L | H | H | L | L | H |
| Paclitaxel versus Other Treatments | | | | | | | |
| Nishina et al (2016) | L | U | H | U | L | L | L |
| Van Cutsem et al (2017) | L | U | H | H | L | L | H |
| Lee et al (2019) | L | U | H | U | L | U | H |
| Hironaka et al (2013) | L | U | H | H | L | L | L |
| Kawase et al (2021) | L | L | H | L | U | L | L |
| Chung et al (2022) | U | U | H | L | U | L | H |
| Fuchs et al (2022) | L | U | H | L | U | L | L |
| Shitara et al (2018) | L | H | H | L | L | L | H |
| Kang et al (2018) | L | U | H | H | L | L | H |
| Shitara et al (2017) | L | U | H | U | U | U | H |
| Docetaxel versus Other Treatments | | | | | | | |
| Ford et al (2014) | L | L | H | H | L | L | L |
| Yi et al (2012) | L | U | H | U | L | L | U |
| Kim et al (2015) | L | U | H | U | L | L | L |
| Lee et al (2017) | L | U | H | H | L | L | H |
| Thuss‐Patience et al (2017) | L | L | L | L | L | L | H |
| Irinotecan versus Other Treatments | | | | | | | |
| Higuchi et al (2014) | L | U | H | L | L | L | L |
| Nishikawa et al 2015 | L | U | H | U | L | L | L |
| Thuss‐Patience et al (2011) | L | U | H | H | L | H | H |
| Sym et al (2013) | L | U | H | U | L | L | L |
| Roy et al (2013) | L | U | H | L | L | L | L |
| Tanabe et al (2015) | L | U | H | L | L | L | L |
| Satoh et al (2015) | L | L | H | L | L | L | L |
| Targeted Treatments and Immunotherapy Treatments | | | | | | | |
| Fuchs et al (2014) | L | L | L | L | L | L | H |
| Tougeron et al (2024) | L | U | H | U | U | L | L |
| Lorenzen et al (2022) | L | L | H | L | U | L | L |
| Yan et al (2022) | U | U | H | H | U | L | L |
| Wei et al (2024) | U | U | H | U | U | L | H |
| **Second- & Later-Line Setting (n=5)** | | | | | | | |
| Lorenzen et al (2022) | L | L | L | L | L | L | L |
| Kang et al (2012) | L | U | H | U | L | L | L |
| Shitara et al (2014) | L | L | H | U | L | L | L |
| Fushida et al (2016) | L | H | H | H | L | L | U |
| Moehler et al (2016) | U | U | L | U | L | L | H |
| **Third- and Later-line Setting (n=2)** | | | | | | | |
| Shitara et al (2020) | L | U | H | L | L | L | L |
| Shitara et al (2018) | L | L | L | L | L | L | L |

**Table 3: Modified PRISMA NMA Checklist of Items to Include When Reporting the results of Network meta-analysis**

| Item | Checklist Item | Reported under which Section |
| --- | --- | --- |
| 1 | Describe the reasons for the evidence review in the context of what is already known, including why an NMA has been conducted. | Yes. The reasons (i.e. current uncertainty of relative efficacy and safety of novel treatments for this indication) are stated in the Introduction section, paragraphs 6 and 7; objective statement. |
| 2 | Specify the study characteristics (for example, PICOs, length of follow-up) and report characteristics (for example, years considered, language, publication status) used to decide the eligibility of studies, giving the reasons for the characteristics used. Clearly describe eligible treatments included in the treatment network and note whether any have been clustered or merged into the same node (with justification). | Yes. Key study characteristics (including PICO, language, study-time period, eligibility criteria) stated at methods 2.1-2.3, PICOS (appendix) & methods 2.5.1). |
| 3 | Describe methods used to explore the geometry of the treatment network and potential biases related to it (for example, if there are important links in the network where the studies are at high-risk of bias). This should include how the evidence base has been graphically summarised for presentation, and what characteristics were compiled and used to describe the evidence base to readers. | Yes. Constructed outcome specific networks (OS, PFS, ORR and Grade ≥3 TRAEs). Feasibility assessed by lines of therapy (2L only feasible). Exchangeability discussed (differences in inclusion/exclusion criteria, biomarker use, prior therapy). Geometry displayed with network graphs (per outcome). Described in methods 2.5.1; Results 3.6 and Appendix Figures. |
| 4 | State the principal summary measures (for example, risk ratio, difference in means). Also describe the use of additional summary measures assessed, such as treatment rankings and surface under the cumulative ranking curve (SUCRA) values, as well as modified approaches used to present summary findings from meta-analyses. | Yes. Stated principal summary measures (HRs for OS and PFS; RRs for ORR, and Grade ≥3 TRAEs; 95% credible intervals reported in the methods 2.6; Results 3.6; figure captions. No treatment ranking/SUCRA presented. |
| 5 | Describe the methods of handling data and combining results of studies for each NMA. This should include but not be limited to: a) handling of multi-arm trials b) selection of variance structure c) selection of prior distributions in Bayesian analyses d) assessment of model fit. | Yes. Descried (Bayesian NMA in GLM framework. OS/PFS: normal likelihood on log-HR scale with identity link. ORR/TRAEs: binomial likelihood with log-link. Priors: Non-informative prior normal distributions (mean 0 and variance 10^6) for treatment-effect parameters specified; half-normal prior (SD 0.5) for random-effects heterogeneity (chosen to reflect between-study heterogeneity). Multi-arm handled within the GLM. Model fit assessed via posterior summaries; analyses run in R.) in methods 2.6 (Data synthesis and statistical methods). |
| 6 | Describe the statistical methods used to evaluate the agreement of direct and indirect evidence in the treatment network(s) studied. Describe efforts taken to address inconsistency when found. | Yes. Described in methods 2.6 (final paragraphs) (Due to few RCTs and absence of closed loops, quantitative inconsistency assessment was not possible. Assumption of exchangeability assessed qualitatively via study/population characteristics; separate networks by line of therapy to reduce structural inconsistency). |
| 7 | Describe methods of additional analyses if done, indicating which were pre-specified. This may include, but not be limited to, the following: a) sensitivity or subgroup analyses b) meta-regression analyses c) alternative formulations of the treatment network d) use of alternative prior distributions for Bayesian analyses (if applicable). | No formal sensitivity analyses, subgroup analyses, meta-regressions, or alternative priors were undertaken because of sparse networks and low event counts; this is acknowledged in the methods 2.6 and discussion. |
| 8 | Provide a network graph of the included studies to enable visualisation of the geometry of the treatment network. | Yes. Network diagrams for each outcome are provided. Figures: Fig 2 (OS), Fig 3 (PFS), Fig 4 (ORR), Fig 5 (TRAEs). |
| 9 | Provide a brief overview of characteristics of the treatment network. This may include commentary on the abundance of trials and randomised patients for the different interventions and pairwise comparisons in the network, gaps of evidence in the treatment network, and potential biases reflected by the network structure (for example, publication bias). | Yes. Described in results 3.1-3.3 and 3.6 and discussion summary (44 RCTs identified; network feasible only in 2L: 8 treatments in OS network, 5 in PFS; 6 in ORR; 5 in TRAEs. Heterogeneity in trial characteristics noted; some treatments absent from certain networks; low events for TRAEs, wide CrIs. |
| 10 | Present results of each meta-analysis done, including confidence/credible intervals. In larger networks, authors may focus on comparisons versus a particular comparator (for example, placebo or standard care). League tables and forest plots may be considered to summarise pairwise comparisons. If additional summary measures were explored (such as treatment rankings), these should also be presented. | Yes. Pairwise results shown as grid (league) tables with 95% CrIs and colour-coded direction of effect for each outcome; full pairwise matrices provided in results 3.6; Figures/Appendix tables. However, no ranking reported. |
| 11 | Describe results from investigations of inconsistency. This may include such information as measures of model fit to compare consistency and inconsistency models, P values from statistical tests, or summary of inconsistency estimates from different parts of the treatment network. | Not applicable. No closed loops and sparse data prevented formal inconsistency testing; this is stated explicitly in methods 2.6; Results narrative |
| 12 | Give results of additional analyses, if done (for example, sensitivity or subgroup analyses, meta-regression analyses, alternative network geometries studied, alternative choice of prior distributions for Bayesian analyses). | Not applicable, no additional analyses conducted due to network sparsity as discussed in methods 2.6 and limitation noted in discussion. |
| 13 | Discuss limitations at study and outcome level (for example, risk of bias), and at review level (for example, incomplete retrieval of identified research, reporting bias). Comment on the validity of the assumptions, such as transitivity and consistency. Comment on any concerns regarding network geometry (for example, avoidance of certain comparisons). | Limitations included inability to build second- and later-line, or third- and later-line networks; incomplete inclusion of all treatments in each outcome network; potential bias from trial heterogeneity; sparse TRAEs; inclusion of pembrolizumab trials not restricted to MSI-H/TMB high; reliance on exchangeability assumption; no quantitative heterogeneity/inconsistency analysis as mentioned in discussion (limitations paragraphs). |

Search Strategy

The clinical search terms for ‘advanced gastric cancer’ and ‘randomized controlled trials’ were modified from the search undertaken in the systematic review and meta-analysis for clinical outcomes of systemic treatments for advanced gastric or gastroesophageal junction cancer by Abderhalden et al., 2023 [1]. Search terms for drugs were obtained from a systematic literature review of health-economic evaluations for systemic treatments for the second- and later-line treatment for advanced and metastatic gastric cancer (Sharma S et al., 2023)[2]. The search terms for the MEDLINE, Embase and the Cochrane library databases are presented below.

| **Embase** | | **N=** |
| --- | --- | --- |
| 1. | 'stomach carcinoma'/exp OR 'stomach carcinoma' | 44,256 |
| 2. | 'stomach cancer' | 124,460 |
| 3. | 'stomach adenocarcinoma' | 20,427 |
| 4. | gastric OR 'stomach'/exp OR stomach OR esophagogastric OR oesophagogastric OR gastroesophageal | 791895 |
| 5. | cancer OR carcinoma OR adenocarcinoma | 5,890,863 |
| 6. | advanced OR metastatic OR metastasis OR recurrent OR unresectable OR inoperable OR incurable OR palliative | 3,111,922 |
| 7. | #4 AND #5 AND #6 | 103,474 |
| 8. | #1 OR #2 OR #3 OR #7 | 200,424 |
| 9. | 'fluorouracil' OR '5 fu' OR 'efudex':ab,ti OR ((keytruda OR pembrolizumab OR mk) AND 3475) OR lambrolizumab OR 'mk 3475' OR ((lonsurf OR trifluridine) AND tipiracil AND hydrochloride) OR 'taxotere' OR 'docetaxel':ab,ti OR 'entrectinib' OR 'rozlytrek':ab,ti OR docetaxel OR paclitaxel OR irinotecan OR (trastuzumab AND deruxtecan) OR (trifluridine NEXT/2 tipiracil) OR lonsurf OR 'taxane' OR 'folfiri':ab,ti OR 'camptosar' OR 'irinotecan':ab,ti OR 'paclitaxel' OR 'taxol':ab,ti OR 'dostarlimab' OR 'jemperli':ab,ti OR 'programmed death 1 ligand 1':ab,ti | 377,170 |
| 10. | 'cyramza' OR 'ramucirumab':ab,ti OR ((enhertu OR 'fam trastuzumab') AND 'deruxtecan nxki') | 2576 |
| 11. | 'camptosar' AND 'fluorouracil' OR ('camptosar' AND 'fluorouracil' AND leucovorin) OR ('camptosar' AND 'fluorouracil' AND 'ramucirumab') OR ('irinotecan' AND 'fluorouracil' AND 'ramucirumab' AND leucovorin) OR ('camptosar' AND 'ramucirumab') OR ('irinotecan' AND 'docetaxel') | 7,982 |
| 12. | ntrk AND gene AND fusion AND positive OR (dostarlimab NEXT/2 gxly) OR 'msi h' OR (dmmr AND tumors) | 5,786 |
| 13. | 'tumor mutational burden' | 10,752 |
| 14. | microsatellite AND 'instability high' | 1,699 |
| 15. | her AND 2 AND overexpression | 4,710 |
| 16. | #9 OR #10 OR #11 OR #12 OR #13 OR #14 OR #15 | 396,384 |
| 17. | clinical AND trial OR 'randomized controlled trial' OR controlled AND clinical AND trial OR phase AND 3 AND clinical AND trial OR phase AND 4 AND clinical AND trial OR randomization OR phase AND 2 AND clinical AND trial OR (phase AND ii OR phase) AND 2 OR 'multicenter study' OR 'single blind procedure' OR 'double blind procedure' OR 'crossover procedure' OR 'placebo' OR 'prospective study' OR rct OR (treble OR triple) AND blind | 5,042,397 |
| 18. | #8 AND #37 AND #38 | 11,153 |
| 19. | #18 AND (2019:py OR  2020:py OR 2021:py OR 2022:py OR 2023:py OR 2024:py) | 2,928 |
| 20. | #19 AND ('article'/it OR 'review'/it) | 2,035 |
| 21. | #20 AND ('article'/it OR 'review'/it) AND [english]/lim | 2,013 |

| **Medline updated** | | N= |
| --- | --- | --- |
| 1. | gastric cancer.mp. or exp Stomach Neoplasms/ or (gastric adj3 (cancer$ or oncolog$ or malignan$ or carcinoma$ or adenocarcinoma$ or tumour$ or tumor$)).ti,ab. | 149,491 |
| 2. | (esophagogastric or oesophagogastric or gastroesophageal).mp. and (cancer or carcinoma or adenocarcinoma).mp. and (advanced or metastatic or metastasis or recurrent or unresectable or inoperable or incurable or palliative).mp. | 4,056 |
| 3. | 1 or 2 | 151,087 |
| 4. | Cyramza.ti,ab,ot,hw. or Ramucirumab.ti,ab,ot,hw. | 1,333 |
| 5. | (Enhertu or Fam-Trastuzumab Deruxtecan-nxki).ti,ab,ot,hw. or 5-FU.mp. or Fluorouracil/ or (Keytruda or Pembrolizumab or MK 3475 or lambrolizumab or MK-3475).ti,ab,ot,hw. or ((Lonsurf or Trifluridine) and (Tipiracil adj2 Hydrochloride)).ti,ab,ot,hw. or (Taxotere or Docetaxel).ti,ab,ot,hw. or (entrectinib or Rozlytrek).ti,ab,ot,hw. | 81,687 |
| 6. | Docetaxel/ or Paclitaxel/ or Irinotecan/ | 49,066 |
| 7. | (trastuzumab adj2 deruxtecan).ti,ab,ot,hw. or trifluridine-tipiracil.mp. or Lonsurf.mp. or leucovorin.mp. or FOLFIRI.mp. or (5-FU adj3 leucovorin irinotecan).ti,ab,ot,hw. or Efudex.mp. or dostarlimab.ti,ab,ot,hw. or DNA Mismatch Repair/ or Jemperli.mp. or Programmed Cell Death 1 Receptor/ or programmed death 1 ligand 1.mp. | 32,633 |
| 8. | (Fluorouracil/ and Irinotecan/) or (Leucovorin/ and Fluorouracil/ and Irinotecan/) or (Fluorouracil/ and Irinotecan/ and Ramucirumab.ti,ab,ot,hw.) | 3,415 |
| 9. | (Leucovorin/ and Ramucirumab.ti,ab,ot,hw. and Fluorouracil/ and Irinotecan/) or (Ramucirumab.ti,ab,ot,hw. And Irinotecan/) or (Irinotecan/ and Docetaxel/) or (NTRK gene fusion-positive.mp.) or ((MSI-H or dMMR tumors).mp.) or (Dostarlimab-gxly.ti,ab,ot,hw.) | 3,102 |
| 10. | 4 or 5 or 6 or 7 or 8 or 9 | 138,284 |
| 11. | Randomized Controlled Trials as Topic/ | 170,284 |
| 12. | randomized controlled trial/ | 613,756 |
| 13. | Random Allocation/ | 107,232 |
| 14. | Double Blind Method/ | 178,659 |
| 15. | Single Blind Method/ | 33,521 |
| 16. | clinical trial/ | 539,956 |
| 17. | clinical trial, phase ii.pt. | 41,315 |
| 18. | clinical trial, phase iii.pt. | 22,741 |
| 19. | clinical trial, phase iv.pt. | 2,499 |
| 20. | controlled clinical trial.pt. | 95,538 |
| 21. | randomized controlled trial.pt. | 613,756 |
| 22. | multicenter study.pt. | 347,146 |
| 23. | clinical trial.pt. | 539,956 |
| 24. | exp Clinical Trials as topic/ | 392,540 |
| 25. | 11 or 12 or 13 or 14 or 15 or 16 or 17 or 18 or 19 or 20 or 21 or 22 or 23 or 24 | 1,600,993 |
| 26. | (clinical adj trial$).tw. | 513,299 |
| 27. | ((singl$ or doubl$ or treb$ or tripl$) adj (blind$3 or mask$3)).tw. | 205,343 |
| 28. | PlaceboS/ | 35,958 |
| 29. | Placebo$.tw. | 256,616 |
| 30. | randomly allocated.tw. | 38,691 |
| 31. | (allocated adj2 random$).tw. | 42,617 |
| 32. | 26 or 27 or 28 or 29 or 30 or 31 | 839,317 |
| 33. | 32 or 25 | 1,984,810 |
| 34. | case report.tw. | 427,350 |
| 35. | letter/ | 1,255,155 |
| 36. | historical article/ | 370,088 |
| 37. | 35 or 36 or 37 | 2,032,469 |
| 38. | 33 not 37 | 1,940,921 |
| 39. | 3 and 10 and 38 | 2,607 |
| 40. | limit 40 to yr="2019- 2024" | 457 |
| 41. | limit 42 to english language | 452 |

|  | Cochrane Central Register of Controlled Trials from 2019 to 29th May 2024 | N= |
| --- | --- | --- |
| 1. | (Stomach Neoplasms or (stomach or gastric) adj3 (cancer* or carcinoma* or tumo?r* or neoplasm*) or (stomach or gastric) adj3 adenocarcinoma* or (gastroesophageal or esophagogastric) adj3 (cancer* or carcinoma* or tumo?r* or neoplasm*) or (gastroesophageal or esophagogastric) adj3 adenocarcinoma*) AND (advance$ or metasta$ or recurr$ or unresect$ or non-resect$ or disseminated or stage 3 or stage III* or stage 4 or stage IV* or spread$ or migration$ or progress$ or invasive or aggressive or "not operable" or untreatable or "not treatable" or secondary or incurable or "not curable") in title abstract keyword - (Word variations were searched) | 3064 |

References

1. Abderhalden, L.A., et al., *Clinical Outcomes for Previously Treated Patients with Advanced Gastric or Gastroesophageal Junction Cancer: A Systematic Literature Review and Meta-Analysis.* J Gastrointest Cancer, 2023. **54**(4): p. 1031-1045.

2. Sharma, S., et al., *Systematic Review of Economic Evaluations of Systemic Treatments for Advanced and Metastatic Gastric Cancer.* Pharmacoeconomics, 2024. **42**(10): p. 1091-1110.
